# Incorporating Existing Network Information into Gene Network Inference

**DOI:** 10.1371/journal.pone.0006799

**Published:** 2009-08-27

**Authors:** Scott Christley, Qing Nie, Xiaohui Xie

**Affiliations:** 1 Department of Mathematics, University of California Irvine, Irvine, California, United States of America; 2 Department of Computer Science, University of California Irvine, Irvine, California, United States of America; 3 Center for Mathematical and Computational Biology, University of California Irvine, Irvine, California, United States of America; 4 Center for Complex Biological Systems, University of California Irvine, Irvine, California, United States of America; 5 Institute for Genomics and Bioinformatics, University of California Irvine, Irvine, California, United States of America; National University of Ireland Galway, Ireland

## Abstract

One methodology that has met success to infer gene networks from gene expression data is based upon ordinary differential equations (ODE). However new types of data continue to be produced, so it is worthwhile to investigate how to integrate these new data types into the inference procedure. One such data is physical interactions between transcription factors and the genes they regulate as measured by ChIP-chip or ChIP-seq experiments. These interactions can be incorporated into the gene network inference procedure as a priori network information. In this article, we extend the ODE methodology into a general optimization framework that incorporates existing network information in combination with regularization parameters that encourage network sparsity. We provide theoretical results proving convergence of the estimator for our method and show the corresponding probabilistic interpretation also converges. We demonstrate our method on simulated network data and show that existing network information improves performance, overcomes the lack of observations, and performs well even when some of the existing network information is incorrect. We further apply our method to the core regulatory network of embryonic stem cells utilizing predicted interactions from two studies as existing network information. We show that including the prior network information constructs a more closely representative regulatory network versus when no information is provided.

## Introduction

Considerable progress has been obtained in the ability to infer gene regulatory networks from gene expression data. The three primary methodologies include probabilistic graphical models [1], information-theoretic approaches [2], [3], and ordinary differential equations (ODEs) [4], [5], see [6]–[9] for reviews of these and other approaches. However using only gene expression data will likely not be sufficient because the noise inherent in the measurements as well as the expense and difficulty to obtain numerous measurements under different experimental conditions implies the inference process on a whole-genome level will always be underdetermined with respect to the amount of data available. Recent techniques attempt to integrate additional data sources or introduce constraints to help guide the inference procedure. Such techniques consider including modeling of environmental and transcription factor interactions [10], [11], incorporating DNA motif sequence in gene promoter regions [12]–[14], combining multiple microarray datasets from the same organism across multiple experiments [15], [16] or from completely different organisms [2], and integrating proteomics and metabolomics [17]. Yet despite these advances, gene network inference remains an extremely difficult problem and new integrative techniques still need to be explored.

One particularly interesting type of data is experimentally determined physical interactions whereby the genes regulated by specific cis-acting transcription factors are identified. These experimental approaches use protocols such as ChIP-chip and ChIP-seq [18], [19] to perform genome-wide measurements, and they have been used to construct putative regulatory networks [20]–[22] under the assumption that binding peaks discovered in gene promoter regions implies regulation of those genes. These protocols are also useful to measure data such as DNA methylation distributions [23], epigenetic state and chromatin structure [24], [25], and transcription factor promoter occupancy [20]. While this data has been used with gene expression data for identification of regulation for a small set of genes [26], [27], there currently is no research utilizing this type of data as part of the genome-wide computational inference of gene networks. This experimental strategy provides high-quality interaction data but is restricted in that the transcription factors must be known in advance and effective antibodies must be available for the ChIP protocol to work, therefore many interactions are missed and only provides a small subset of the regulatory network. However, these interactions can be utilized as a priori network information to help guide inference procedures. Probabilistic approaches can incorporate this existing network information through prior distributions [28], [29], but these techniques are computationally expensive. ODE methods are more computationally tractable, however there is no existing research that shows how to systematically incorporate prior network information. We fill that void in this article by extending the ODE methodology into a general optimization framework that incorporates gene expression data with existing network information. Our approach continues upon earlier work by also utilizing regularization parameters to encourage network sparsity, and we show that various types of experimental data can be formulated into the framework. We prove that our estimator is asymptotically root-n consistent with the estimated weights converging to the true weights at a rate of 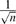, where *n* is the number of data observations, and that there is a corresponding probabilistic interpretation which is also asymptotically root-n consistent.

We test our method on simulated network data and show that existing network information improves performance, overcomes the lack of observations, and performs well even when some of the existing network information is incorrect. We demonstrate the applicability of our framework to real biological data by inferring the core regulatory network for embryonic stem cells. We utilize predicted interactions from two experimental studies, each using a different experimental technique, as existing network information, and we show that including the experimental network data constructs a more closely representative network versus when no information is provided.

## Methods

Gene network inference based on ordinary differential equations (ODEs) describes gene regulation as a function of other genes:
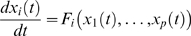
(1.1)where 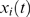 is the concentration of mRNA for gene *i* measured at time *t*, 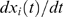 is the rate of change for the mRNA concentration of gene *i*, and *p* is the number of genes. Each function 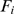 represents all of the various factors that affect the amount of mRNA for gene *i* including processes such as transcription rate, degradation, post-transcriptional modifications, translation rate, etc. This representation indicates a causal interaction versus a conditional probability as with statistical approaches, but does not necessarily imply a physical interaction or a direct relationship as proteins, metabolites, transcription factor binding, and other regulatory processes are not explicitly represented. The advantage of this dynamical system representation is that the model can be expanded to include any of these more detailed interactions. Though this introduces additional flexibility as it adds more parameters to the model, little data is available for these intermediate processes, and the functional form is generally not known. Furthermore, the system may be amenable to analysis to deduce properties such as existence of steady-state solutions, bi-stability and sensitivity analysis of parameter values; and numerical simulation can be performed for quantitative prediction and validation.

Variations exist with the exact formulation depending on whether the available expression data is time-series or steady-state measurements, the assumed experimental noise model, and the particular function form for 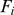 that is chosen. For current simplicity of presentation we will take the form that approximates the gene regulatory network with a linear system of equations such as used by Gardner et al. [5] and expanded upon by others [4], [16], [30]; however we will show in later sections how different forms of data can be included as well as non-linear functions. The model considers a set of external perturbations *u* that have been applied to one or more gene resulting in the following set of linear ODEs:
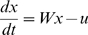
(1.2)where W is a 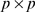 matrix containing the interaction coefficients and constitutes the network model to be inferred. Given *n* observations, 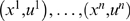, of the mRNA concentrations for *p* genes and their perturbations, and under the assumption the observations are made at steady-state 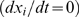, inferring *W* in Eq. (1.2) can be expressed as a least-squares minimization problem:
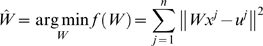
(1.3)


### Regularization

For 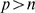, the linear system is underdetermined. Gardner et al. [5] with their network identification by multiple regression (NIR) algorithm argue that assuming a maximum of *k* incoming connections serves to transform the problem into an overdetermined system, and makes it robust to measurement noise and incomplete data. However, this restriction does not reliably prevent overfitting and all genes tend to have exactly *k* incoming connections to minimize the linear regression error, regardless of whether all those connections are valid. Computationally, the NIR algorithm has to run 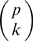 multiple linear regressions for each gene which becomes intractable for large *p* and modest values for *k*. Even if there are enough observations, regression tends to use as many genes as possible to explain the data and thus overfits by including all *k* network connections. A more appropriate methodology is one based on sparsity. Genes should only have enough connections to predict their expression data without overfitting, and genes should be allowed to have differing number of connections to properly reflect the underlying network structure implied by the data. Various regularization techniques have been introduced to prevent overfitting including ridge regression [31], LASSO [32]–[34], and elastic net [35]. Ridge regression uses an L_2_-norm constraint to maintain the best predictors, but it does not encourage sparsity and is not necessarily the most parsimonious model. The LASSO (least absolute shrinkage and selection operator) method adds an L_1_-norm constraint; this constraint tends to produce connection coefficients that are exactly zero, and thus acts to enforce parsimony. Elastic net combines both of these constraints. Gustafsson et al. [4] and the Inferelator [10] both use LASSO and provide evidence that it selects parsimonious models. We consider using LASSO as the basis of our algorithm to enforce network sparsity and will enhance it to include existing network information, so the minimization problem becomes:
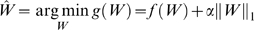
(1.4)where 
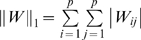
and 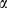 is a positive parameter that enforces the level of sparsity in the gene network. The parameter 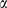 is learned through cross-validation with larger values for 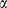 producing a more sparse matrix while 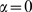 corresponds to the standard least-squares regression problem.

### Incorporating existing network information

Existing network information can be incorporated into the minimization problem by adding an additional constraint for connections in the network. Given a 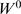 matrix with positive entries 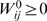 indicating the lack of interaction for gene 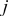 on gene *i*, the problem becomes:
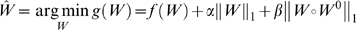
(1.5)where 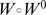 denotes the entry-wise product between matrix 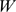 and 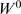. This adds a penalty to edges in 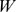 that do not exist in 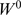, making those edges less likely to be included. Notice that this formulation does not force edges provided by the existing network information to be included in the resultant network; instead those edges are just not penalized with 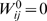 which will make them more likely to be picked over other edges. This allows the optimization to still pick a different network structure if it fits the data better. The strength of the penalty is determined by a positive parameter 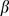, which is learned through cross-validation. If the existing network information is not beneficial to reducing the error of the inferred network model, then cross-validation will set 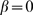 to eliminate the penalty. However 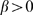 signifies that the existing network information is beneficial.

### Optimization framework

We introduce a general optimization framework for various types of gene expression data that incorporates sparsity and existing network information. This formulation encompasses the standard least-squares problem as in Eq. (1.3), yet it is flexible enough to handle gene-specific problem alterations such as those required for certain kinds of gene expression perturbation data. Let 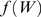 be a quadratic function of the 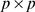
square matrix 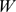, defined in the form:
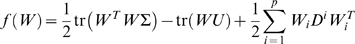
(1.6)where 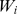 denotes the *i-*th row vector of the matrix 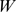. Matrices 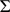 and 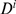 are symmetric and positive definite, that is 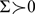 and 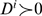 for all 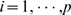. Under this definition 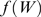 is a convex function of 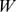. Our goal is to find 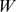 that minimizes 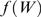 subject to sparsity constraint and existing network information:
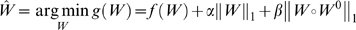
(1.7)


We simplify the notation to the following:
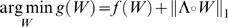
(1.8)by defining a 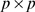 matrix 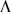 that combines the two parameters:
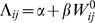
(1.9)


We use a coordinate descent algorithm to solve this optimization problem for a given 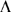 matrix [36]. The algorithm iteratively updates each 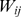 matrix entry until 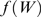 converges to its minimum value; convergence is guaranteed by the convexity of the function and the additivity of the L_1_ regularization term [37]. The derivate of 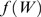 with respect to 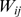 is:
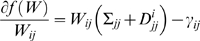
(1.10)where 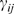 is independent of 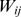 and is defined to be:
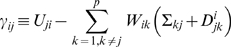
(1.11)


Now consider the derivate of 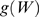 in Eq. (1.8) with respect to 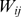:
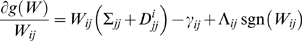
(1.12)where
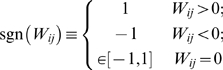
(1.13)


Therefore the update rule in the coordinate descent algorithm is:
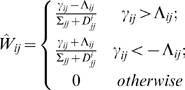
(1.14)The resultant network model 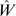 is dependent upon the values used for the two parameters 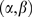, so we use cross-validation to find values that provide the minimum total testing error. K-fold cross-validation is common practice; however there tends to be few observations for gene expression data, so we have used leave-one-out cross-validation which puts just one observation into the testing set. Note that if all 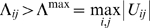 then all matrix entries 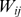 will be constrained to be zero, therefore:
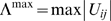
(1.15)This provides bounds 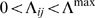 that need to be searched. We perform an exponential search starting from 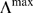 and going down, using the 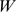 matrix as a warm start from one value to the next. In fact, 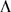 is comprised of two parameters so a matrix of 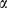 and 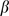 values is constructed and the minimum error for the parameter pair is chosen.

### Perturbation gene expression data

Suppose we are given a set of 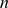 observation, 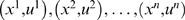, representing the activities of genes 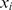 in the network after input perturbation 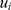, for 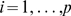. We infer the network connections 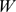 between the genes by minimizing the following error function:
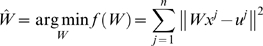
(1.16)The error function can be rewritten as:
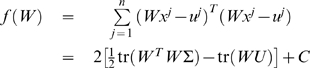
(1.17)where *C* is a term independent of *W* thus dropping out of the minimization, and the matrices 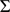 and 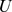 are defined to be:
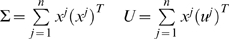
(1.18)Thus standard gene expression perturbation experiments fit in the optimization framework as described in Eq. (1.6) with 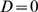.

### Modified formulation for different perturbation experiments

The formulation as described in Eq. (1.3) requires a measurement of the external perturbation distinct from the expression values of the perturbed genes, which might not be available for all types of experiments. We propose an alternate formulation where we consider genetic perturbations 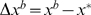 away from the gene expression of the wild-type gene network 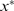. We show in the next sections that we can still express the system as a least-squares minimization problem in the same form as Eq. (1.6) and use our optimization framework to find the best network model to explain the perturbation.

#### Null mutant gene expression data

Null mutant experiments remove or prevent a gene from being expressed; the simplest form is to knock out one gene per experiment then measure the steady-state expression values for the 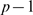 other genes. Suppose we are given 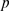 observations where a single different gene 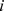 is removed in each observation. Denote the observed steady state by the vector 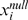, for all 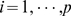 after gene *i* is removed. The perturbation of 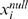 away from the wild-type steady state is:
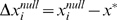
(1.19)We infer the connection model and strengths between the genes by minimizing the following error function:
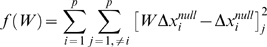
(1.20)Gene 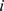 cannot be used to predict itself for the observation when gene 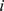 is removed, so the error function indicates this by excluding gene 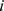 for observation 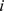. We reformulate the error function and cast into Eq. (1.6) where we have defined:
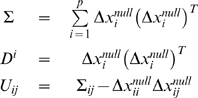
(1.21)


#### Heterozygous knockdown gene expression data

Heterozygous knockdown experiments remove one of two copies of a gene; a series of experiments might knockdown one gene per experiment then measure the steady-state gene expression values for all 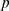 genes. Suppose we are given 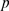 observations where a single different gene 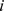 is knocked down in each observation. Denote the observed steady state by the vector 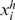, for all 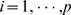 after removing once copy of gene *i*. The perturbation of 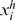 away from the wild-type steady state is:
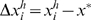
(1.22)


However for the experiment with gene 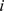 knocked-down, only one copy of gene 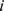 can contribute so we denote the perturbation of the other copy of gene 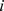 by:
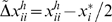
(1.23)We infer the connection model between the genes by minimizing the following error function:
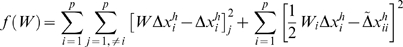
(1.24)We can reformulate the error function and cast into Eq. (1.6) with:
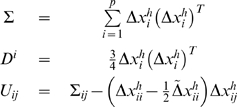
(1.25)


### Time-series gene expression data

Time-series gene expression data has also been utilized in ODE methodology. The basic idea is to no longer assume the system has been measured at steady-state 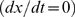, and use the time-series data as an approximation to the derivative. We can still consider perturbations to the system but we should take into account that dynamics of different genes operate on different time scales. We are given a set of trajectories, each from a different initial perturbation, along with *n* observations for all genes at unit time intervals 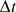 as the system relaxes back to the steady state. The final measurement is not necessarily the steady state. We consider each trajectory as a time sequence, 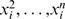, for each gene *i*. We have a linear system of the form:
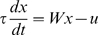
(1.26)The derivative can be estimated in a number of ways, but we will consider here the Mean-Value Theorem approximation:
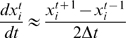
(1.27)The problem is formulated as a least-squares minimization problem and we use our same optimization framework as before:
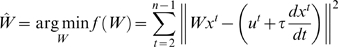
(1.28)


However, we do not know 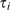 for each gene so we will need to learn it iteratively with *W*. Assume that we have a reasonable initial value for 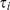, we first calculate 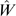 then optimize Eq. (1.26) for each 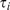:
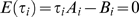
(1.29)where 
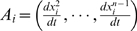
 and 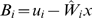. The optimal solution for 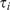 is:
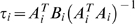
(1.30)Use the determined 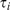 to recalculate the derivative entries in Eq. (1.28) and compute a new 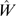, repeat this iteratively until convergence.

### Nonlinear functional form

Consider just a single gene 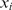 for Eq. (1.3) where we have a nonlinear differentiable response function 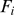 and we want to find:
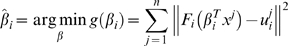
(1.31)where 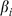 are the interaction coefficients for gene *i*, essentially corresponding to row *i* of the network model *W*. To solve the problem, we first find a quadratic approximation of the objective function around an arbitrary point 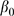:
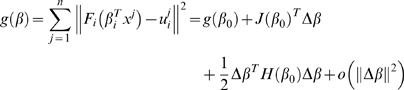
(1.32)where 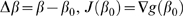 is the gradient of *g* at 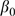, and 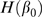 is the Hessian matrix. Specifically, we have:
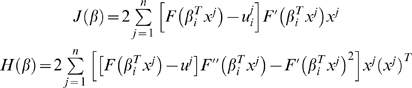
(1.33)Thus, around 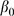, the problem fits into our convex optimization framework and can be solved with the same coordinate descent algorithm:
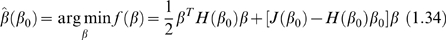
(1.34)


In summary, we propose the following algorithm to independently find the set of interaction coefficients for each gene:

1. Randomly choose 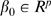.
2. Find 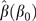 using Eq. (1.34) and the coordinate descent algorithm.
3. Perform line search: find 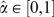 such that
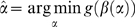
where 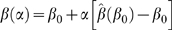.
4. Set 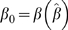 and go to Step 2) if 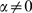.

### Asymptotic properties

Consider Eq. (1.16) with a matrix 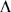 of non-negative entries and a parameter for L_1_-norm regularization:
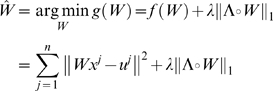
(1.35)This equation is equivalent to:
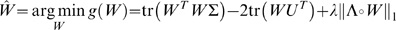
(1.36)with matrices 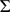 and 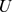 as defined in Eq. (1.18).

Consider the following noise model:
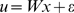
(1.37)where the noise term 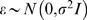 follows normal distribution with a fixed variance.

### Theorem 1

*If* 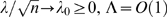, *and*

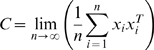
(1.38)
* is non-singular, then when* 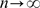 *,*

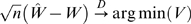
(1.39)
*where*

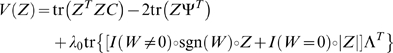
(1.40)
*and* 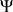 *is a random matrix with normal distribution of mean 0 and covariance* 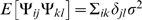 *for all i, j, k, l.*

Proof of the theorem is provided in Appendix S1. This theorem suggests that the estimate is root-n consistent with the estimated 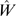 converging to the true W at a rate of 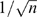 when the penalty term is reduced at a rate of 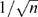. The root-n consistency property is similar to the asymptotic property of the Lasso estimator first described by Knight and Fu [38].

#### A special case: orthogonal design

In this section, we show how prior network information can aid us in network inference with a special case where we can analytically solve for the error rate. Consider the case of an orthogonal design: 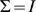. Different entries of W decouple and have the optimal value in the form of a soft-threshold function:
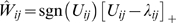
(1.41)


Now consider a noise model of 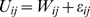 where 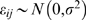 for all i, j. We assume W is sparse with 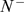 zero entries and 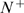 non-zero entries. Note that 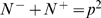. Denote 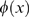 the density of a standard normal distribution and 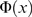 its cumulative distribution. The probability of 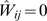 is:
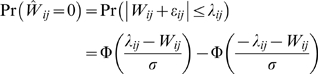
(1.42)If 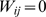, the probability of misidentifying 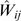 as non-zero is:
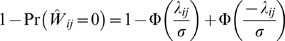
(1.43)The overall error rate of misidentifying non-zero entries as zero and zero entries as non-zero is:
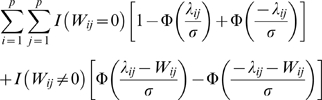
(1.44)For simplicity of discussion, assume 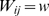 for all non-zero entries and 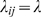 for all i, j. Then the error rate is:
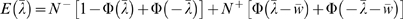
(1.45)where 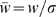 and 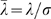. When 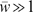, the optimal 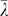 that minimizes 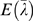 is:
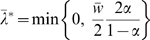
(1.46)where 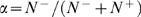 is the proportion of zero entries, or the sparsity of the network. The optimal regularization 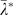 can be derived through cross-validation in real applications if the number of observations is sufficiently large.

Next consider how to incorporate the existing network information (potentially incorrect). Suppose we are provided with the information that a subset of the connections is zero. For these connections, we increase the regularization parameter 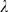 to 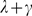 with 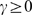. Suppose among the subset, 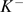 connections are indeed zero and 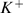 connections are actually non-zero. In summary, we have 4 groups of connections: 1) 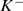 with true zero connection strength and parameter 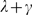; 2) 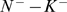 with zero connection strength and parameter 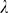; 3) 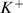 with non-zero connection strength and parameter 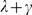; and 4) 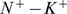 with non-zero connection strength and parameter 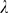. The total error rate is then:
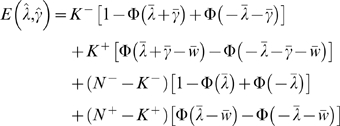
(1.47)where 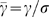. Similarly, if 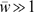, the optimal 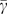 is:
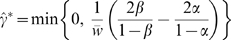
(1.48)where 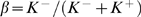 is the sparsity in the subset. Note that 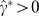 if and only if 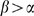. This suggests that by adjusting the parameter 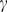 through cross-validation, we should be able to decrease the prediction error rate if the sparsity of the subset is lower than the sparsity of the entire network. On the other hand, if 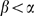, this means that the prior information on the subset is actually worse than a random guess. In that case, the cross-validation should set 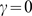 thus ignoring the prior information.

### Probabilistic Interpretation

There is correspondence of the least-square minimization model as described above with a probabilistic interpretation using Gaussian random fields to model the interaction between genes. Under this model, we assume the distribution of gene expression values are described by a multivariate normal distribution with mean 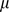 and covariance matrix 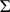. We are interested in the inverse of the covariance matrix, 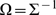, which encodes conditional dependency between two genes conditioned on all others, and therefore it contains information on the connectivity between genes. Our goal is to estimate 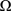 from a set of observations 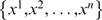 where 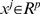 is a vector with dimension *p*, and we assume each 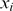 is standardize with mean 0. Then the log likelihood for observing the data is:
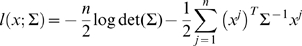
(1.49)up to a constant difference. After standardizing the data, we can rewrite the log likelihood as a function of 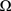:
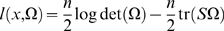
(1.50)where 
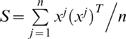
 is the empirical covariance matrix. The inverse covariance matrix 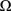 can be estimated by maximizing this log likelihood function. Non-zero entries, 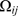, indicate the existence of a connection between gene *i* and *j*. Also note that different from the linear network model, the probabilistic model infers a network with undirected edges as it is making a statement about conditional dependency between two genes. This problem can be solved using several convex optimization algorithms including interior point methods [39] and coordinate descent [34].

When 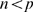, *S* is singular and the solution is underdetermined. Even when 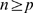, we will likely overfit the data using Eq. (1.50) if *n* is not large. A solution is to add a regularization term to the log likelihood function. In fact, we can incorporate the same L_1_-norm sparsity constraint that we did for the ODE model, thus providing us the capability to both enforce a parsimonious model as well as introduce existing network information. Given a matrix 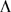 of non-negative entries and a single non-negative parameter 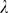, we can formulate the following minimization problem:
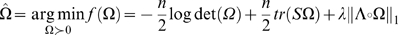
(1.51)where 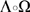 is the component-wise product of the two matrices.

Similar to the linear network inference described in Theorem 1, we can prove (provided in Appendix S1) that the estimated 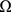 converges to the true 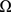 at the rate of 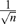 as 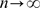 thus showing that the estimate is still root-n consistent.

### Theorem 2

*If* 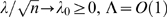, *and* 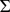 *is non-singular, then*

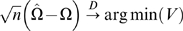
(1.52)
*where*

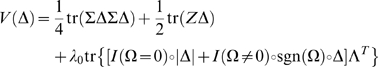
(1.53)
*and Z is a random matrix with normal distribution of mean 0 and covariance* 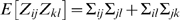 *for all i, j, k, l.*

## Results

### Simulation Results

We generated a set of random linear network models to test the utility of our optimization framework and to characterize the effect of providing existing network information. Each network contains 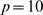 nodes with 2–3 uniform randomly selected incoming edges for a total of exactly 25 edges in the network; a weight for each edge was randomly drawn from the normal distribution 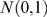. We verified that the generated network, 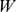, was not singular with a valid inverse then generated a random perturbation matrix, 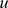, with 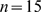 experiments (or observations) for all 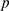 nodes. Each random response value was drawn from the normal distribution 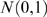 and the observation matrix, 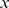, was calculated:
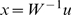
(1.54)


Experimental noise was added to both the perturbation and observation matrices. The noise for the perturbation matrix was drawn from 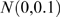 and for the observation matrix was drawn from 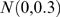. The larger standard deviation for the observation matrix signifies the additive noise for 2–3 incoming edges from the perturbations.

For our experiments utilizing existing network information, we considered the simplest network information that can be provided, the existence of a directed edge going from one gene to another as a boolean value. The set of existing edges is provided as a boolean network 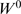 to our algorithm where an entry 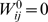 indicates a directed interaction from gene 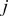 to gene 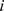 and thus is not penalized, while 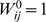 for all other edges.

#### Fewer observations decreases prediction performance

**Figure 1:**
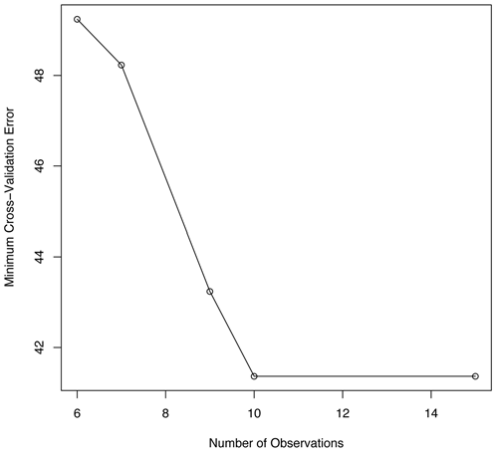
Minimum Cross-Validation Error given Number of Input Observations. The minimum error for the best fitting model as determined by cross-validation and averaged for five simulated linear network models. The network models have ten nodes, so once there are enough observations then the error stabilizes; however the error increases as less observations are provided to the inference algorithm.

One of the key challenges with inferring gene networks from gene expression data is the relatively few observations available compared to the large number of genes. This has a direct effect on how well an inferred model can predict the observed data as illustrated by Figure 1. While an underdetermined linear model can be constructed to fit the data exactly, this is clearly overfitting and cross-validation more correctly specifies the best fitting model. Figure 1 shows how when the number of observations decrease then the error increases for the best fitting model as determined by cross-validation. Furthermore, when the number of observations is greater than the number of variables, then the error stabilizes.

#### Existing network information overcomes fewer observations

**Figure 2:**
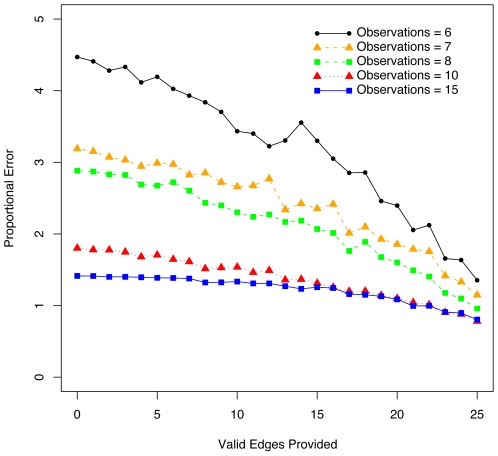
Error Decreases with More Valid Edges Provided as Existing Network Information. Going from zero edges to the fully correct network, randomly selected valid edges are provided as existing network information. The cross-validation error for five simulation runs for five randomly generated linear network models is averaged and plotted as proportional error versus the number of valid edges provided. Because we add experimental noise to the observations, the amount of error varies with the number of observations. Therefore to cancel this bias we calculate a proportional error, which is the minimum cross-validation error averaged across the simulation runs divided by the minimum least-squares error obtained linear regression.

We tested how providing existing network information could overcome the lack of observations by running our algorithm on a set of five randomly generated linear models. We systematically provided more valid edges to the algorithm going from zero edges up to the fully correct network. The results can be seen in Figure 2. For each of the five randomly generated linear network models, we ran our algorithm five times with a different set of edges randomly selected from the correct network. The cross-validation error results are averaged over the 25 total simulation runs for each number of valid edges provided as existing network information.

Figure 2 clearly illustrates that the error decreases as more valid edges are provided to the algorithm. An interesting observation is that the rate of decrease is significantly more for fewer observations; this indicates that each valid edge has a more important role in inferring the correct network model when the total information available is scarce. Therefore, even providing just a few valid edges can substantially improve the inference process. Furthermore note that the variation is greater for fewer observations, we could have made these curves smoother by running more simulations, but the variation illustrates another key point that not all valid edges are equal in their ability to reduce error. Some edges are more important than others, though which edges is typically not known beforehand.

#### Providing incorrect network information does not hurt prediction

**Figure 3:**
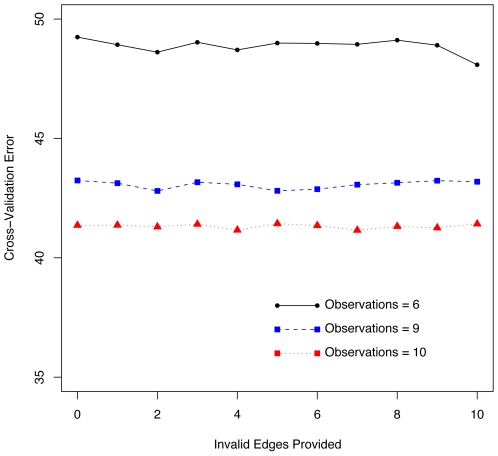
Invalid Edges Does Not Affect Performance. Randomly selected invalid edges provided as existing network information does not affect the minimum error for the best fitting model as determined by cross-validation and averaged for five simulated linear network models.

While we showed in the previous section that providing correct edges as existing network information helps prediction, what about if we give the algorithm incorrect edges? Figure 3 shows the results. If we only provide invalid edges, it does not significantly hurt performance. The reason is apparent if we consider the constraint added to the optimization problem for incorporating network information. If the existing network information provided to the algorithm does not help to reduce the error, then cross-validation will determine the best minimum error is obtained by setting 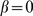, essentially ignoring the network information. The algorithm then performs equally as well as when no network information is provided.

#### Combination of valid and invalid network information still performs better than providing no network information

**Figure 4:**
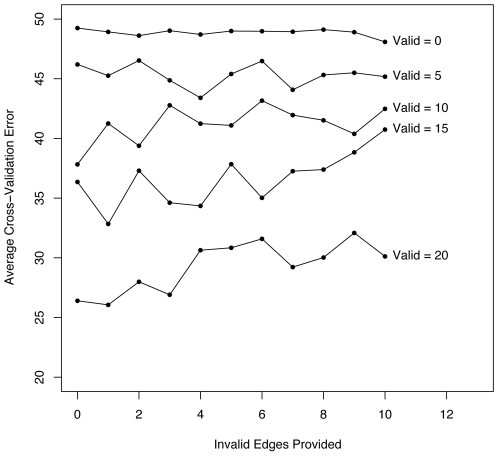
Mixture of Valid and Invalid Edges. Randomly selected valid and invalid edges provided as existing network information still performs well as determined by cross-validation and averaged for five simulated linear network models.

It would not generally be the case that the provided existing network information is completely correct; it is more likely that it contains a mixture of valid and invalid edges. Figure 4 shows that our algorithm still performs well in this mixed situation. When the majority of the edges are valid then adding invalid edges increases the error but not significantly enough to detract from the usefulness of the valid edges. Even when there are only a few valid edges compared to the number of invalid edges, then the algorithm is able to still utilize those valid edges. In all cases, providing existing network information, even with some invalid edges, performs better than providing no network information.

### Biological Results

The discovery that introduction of transcription factors into mouse and human somatic cells is sufficient to induce a pluripotent stem cell fate has generated considerable excitement [40]–[46]. Further experiments have been performed to elucidate the core transcriptional network involved with maintaining pluripotency including correlation of transcription factor binding data with gene expression [21], ChIP-seq [22] and biotin-mediated ChIP [20]. While such experiments suggest potential gene regulatory interactions, they do not provide definitive evidence because not all of the detected interactions may be functional [47]. However such data constitutes prior network information we can provide to our gene network inference procedure, and in this section we will compare an inferred network that includes this prior network information versus a network when no information is provided.

**Figure 5:**
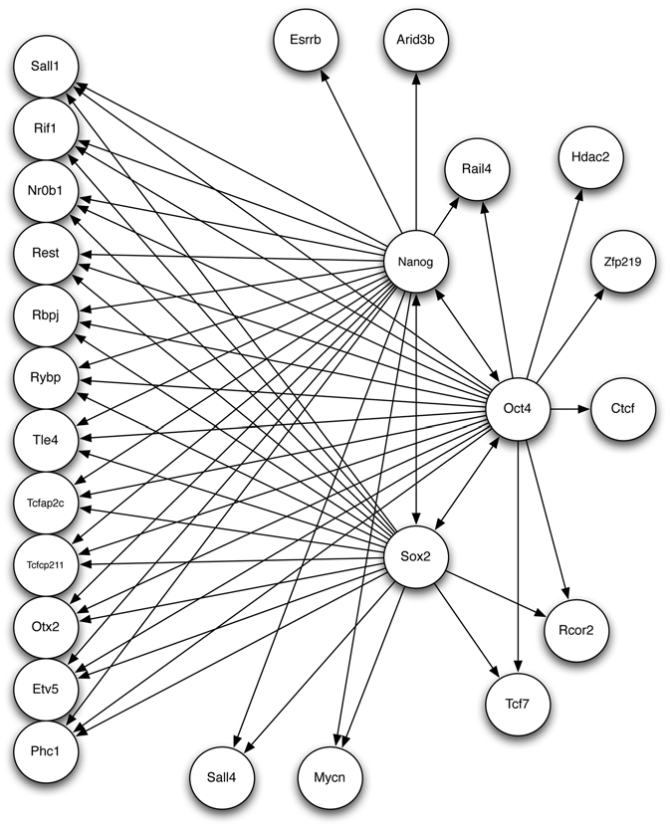
Experiment Transcriptional Network for Mouse Embryonic Stem Cells. Combining the hypothesized interactions obtained through experiments by Zhou et al. [21] and Kim et al. [20] forms prior network information to be provided to our gene network inference algorithm. There are a total of 49 genes but only the 25 genes regulated by the three core factors, Nanog, Oct4 and Sox2, are shown here.

We focus on the 49 total genes that are included in the regulatory networks constructed by Zhou et al. [21] and Kim et al. [20]. In Figure 5 we show this combined network for three core transcription factors (Nanog, Oct4 and Sox2) and the genes they are hypothesized to regulate. While this figure only shows 25 genes, we performed the computational analysis for all 49 genes (provided in Supplemental Text S1) but restrict our discussion for clarity to these three core factors. What can be seen from Figure 5 is that the experimental data suggests cross-regulation between all three factors, a larger set of genes co-regulated by all three factors, and a few genes regulated by one or two of the core factors. We use the gene expression data from Ivanova et al. [48] which consists of a time course set of expression values, however we utilize just the final time points which most closely resemble steady state conditions for a total of 16 observations.

**Figure 6:**
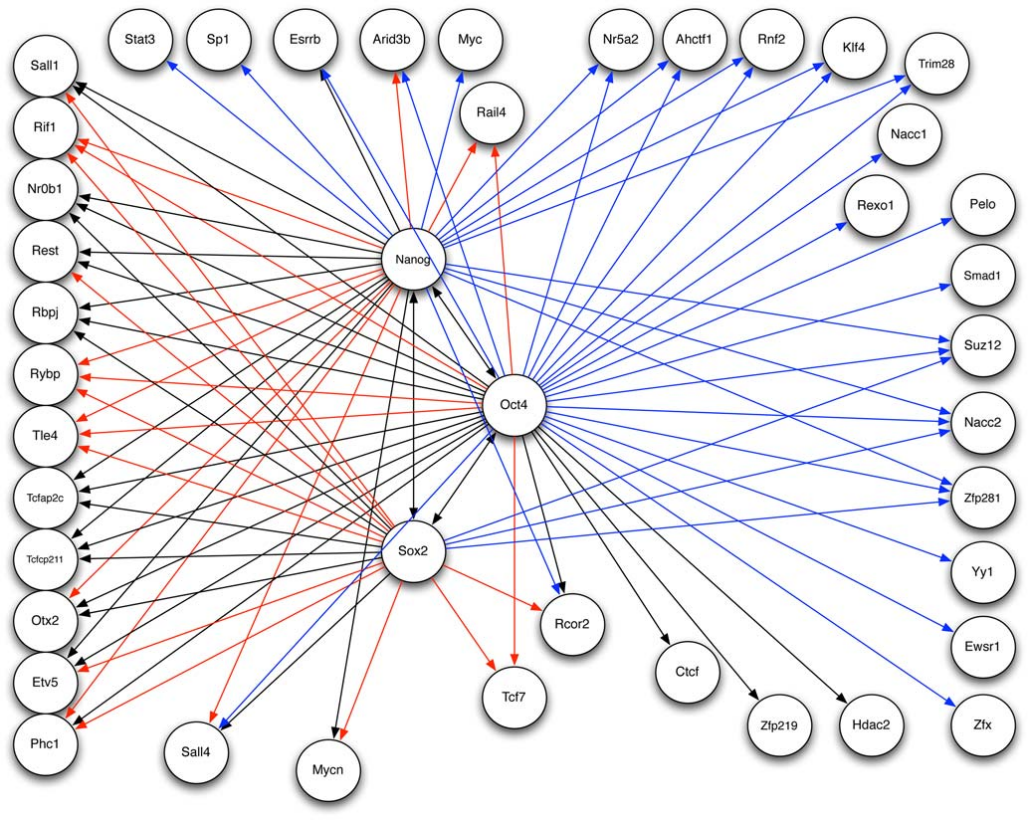
Inferred Network with Prior Information. The gene regulatory network learned through cross-validation with the experimental network provided as existing information. This figure shows just the interactions predicted for the three core factors, Nanog, Oct4 and Sox2. Black edges indicate the inferred interaction matches the experimental network, a red edge indicates an interaction in the experimental network not predicted in the inferred network, and blue edges are new interactions predicted in the inferred network.

**Figure 7:**
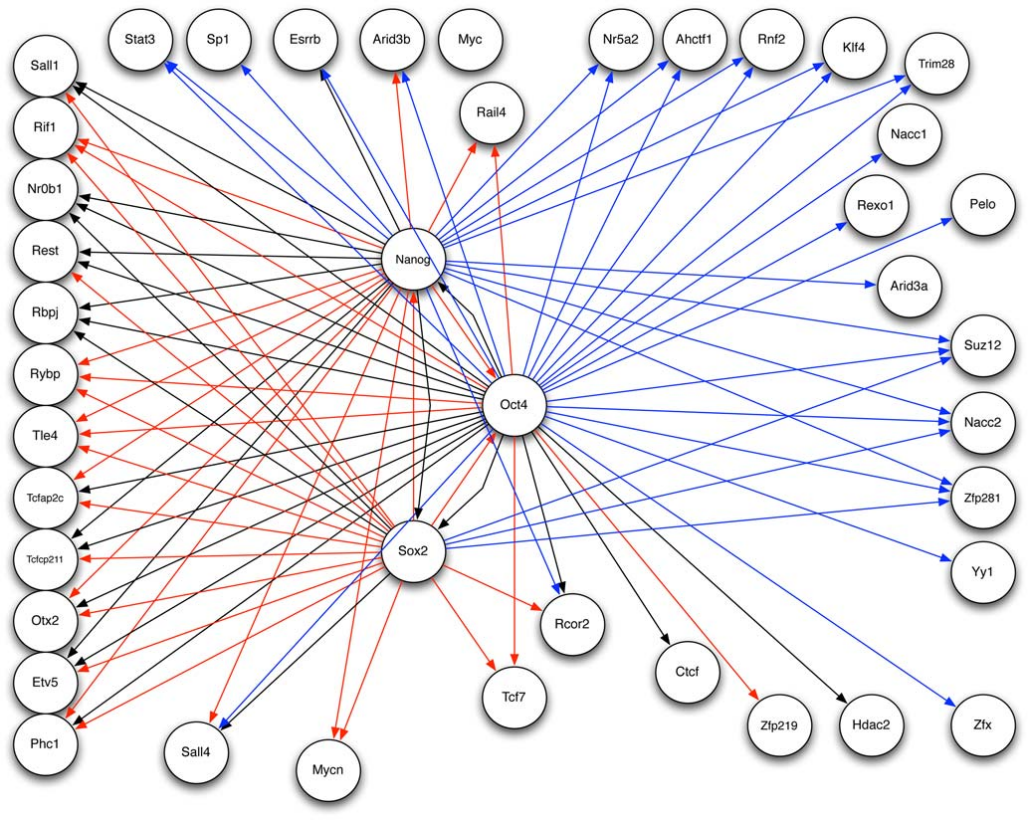
Inferred Network without Prior Information. The gene regulatory network learned through cross-validation with no prior network information provided. This figure shows just the interactions predicted for the three core factors, Nanog, Oct4 and Sox2. Black edges indicate the inferred interaction matches the experimental network, a red edge indicates an interaction in the experimental network not predicted in the inferred network, and blue edges are new interactions predicted in the inferred network.

Using leave-one-out cross-validation, we find the values for the 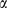 (sparsity) and 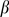 (prior network) parameters for each gene that minimizes the total testing error. Figure 6 shows the resulting network inferred by our algorithm when given prior network information. For all three core factors, the algorithm gave more weight to the prior network over just the sparsity constraint; Nanog 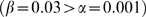, Oct4 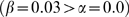 and Sox2 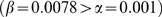. This was not true for all genes, with 23 of the 49 genes having an 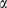
value less than or equal to the 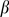
value; the full set of learned parameter values are provided in Supplemental Text S1. We also used cross-validation to learn a network without prior information, so only finding the best 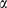 parameter to minimize the testing error. The resultant network is shown in Figure 7.

For both Figure 6 and 7, color is used to illustrate the comparison between the prior network in Figure 5 with the inferred network. Black edges indicate the inferred network predicted the same interaction as in the prior network, red indicates a prior network interaction not predicted in the inferred network, and blue edges are new interactions predicted in the inferred network. While the two inferred networks appear similar, there are significant differences. Overall the inferred network with prior information maintained more edges in correspondence with the prior network keeping 34 edges while the network without prior information kept only 25 edges. Both networks added about the same number of new interactions with 33 for the network with prior information and 32 for the network without prior information. Most notably the network with prior information maintained the co-regulatory interactions between the three core factors while this is lost in the other network.

Closer inspection of the interactions (red edges) present in the experimental network but not in the inferred networks show that for some genes all interactions with the core transcription factors were not predicted. These genes are Rif1, Rybp, Tle4, Tcf7 and Rail4, and these represent the majority of interactions missing in the inferred networks. There are several possible explanations. One is anomalous expression data for the genes, however basic statistical analysis of the data shows the mean expression values for each gene is much greater than its standard deviation which indicates the expression data is not dominated by noise. A second possibility is that the interaction is sufficiently non-linear so a linear approximation fails to capture the connection, but an alternative explanation is that the inferred network failed to confirm a functional interaction as suggested by the experimental network. These five genes are new predictions in the experimental network, and there is little or no experimental confirmation so the lack of correspondence draws into question the validity of those interactions. While we have not checked all interactions, it is encouraging to note that some interactions shared between the experimental and inferred networks include genes known to be important for pluripotency such as Sall4 [49] and Esrrb [50] as well as new inferred interactions such as Oct4 to Yy1 [51]. The combination of gene network inference coupled with the incorporation of existing network information provides a stronger framework for predicting true functional interactions while also doing a better job of excluding false positives.

## Discussion

We have taken the ODE methodology for inferring gene networks from gene expression data and extended it to incorporate a priori network information, such that might be obtained from additional biological data like ChIP-chip or ChIP-seq experiments. We have devised a general optimization framework and algorithm that can be applied to gene expression data for a variety of experiments such as perturbation, null mutant, and heterozygous knockdown. Though the focus of the presentation has been on linear ODEs, we have shown that a quadratic approximation of nonlinear functions can be used in the context of the optimization framework. While use of regularization parameters to encourage sparsity in inferred gene networks has been previously reported, we believe this is the first research that utilizes such regularization to integrate existing network information into the inference procedure. We have tested our algorithm on simulated linear network model data and showed that existing network information improves performance, can overcome the lack of observations, and performs well even when some of the existing network information is incorrect.

The method that we use to incorporate existing network information does not force edges to be present in the inferred network model. The penalty actually serves to prevent other edges from being included in the network, so it very much acts as a soft constraint. The algorithm is free to pick other edges if they ultimately fit the data better and cross-validation will insure this by appropriate computation of the parameter value. Though we do not describe the specifics, it is possible to force an edge to be included in the final network. This can be done by removing all of the L_1_-norm regularization during the calculation for that specific edge. The coordinate descent algorithm in our optimization framework will then perform the standard least-squares calculation for that edge. Another advantage of our framework for existing network information is that a “confidence” value can be provided for each edge, thus penalizing some edges more or less, by using different values in the 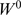 matrix. Furthermore, edges can be effectively removed from the network just by setting their 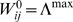.

Our presentation of the optimization framework indicates that there is a single value for the 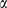 and 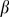 parameters for the whole network. However, the interaction coefficients for each gene can be considered independent from the other genes. This means that it is possible to have a separate 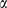 and 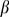 for each gene. The algorithm would still use cross-validation to calculate the parameter values but it would be done separately for each gene. This introduces more parameters into the model so it has a greater chance of overfitting the data but it also has the advantage of allowing the framework to more selectively utilize existing network information, especially in the situation where the information is good for one gene but poor for another gene. With a single parameter value, the optimization framework has to balance good and bad information for the whole network. Splitting the calculation also makes the algorithm more amenable to parallel computation because each gene is optimized independently and can be performed by separate programs without any synchronization. The final W matrix is constructed by merging the results of the individual genes together. Even with single values for 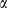 and 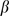, parallelization can still be utilized either for individual genes and/or for cross-validation, but synchronization is required at intermediate steps.

Computationally, a single optimization run is fast and efficient. For both the simulated and biological networks, a single optimization finished quickly in just a few seconds. What can actually take considerable time is cross-validation to learn the best 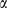 and 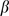 parameter values. Here one constructs a two-dimensional grid of parameter values, a lower-triangular matrix as entries above the diagonal correspond to solutions with 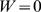. Then for each grid entry a set of optimizations is run to infer a gene network for a subset of observations as well as calculate the cross-validation error. For the biological data with 16 observations, that entails 16 optimization runs per grid entry and with a resolution of 50 intervals for each parameter for a total of 1275 grid entries; inferring the complete gene network in Figure 6 requires over 20,000 optimization runs. Even so this takes only about 1.5 hours on a standard desktop Mac computer. Scaling to genome-wide networks with thousands of genes is certainly a challenge but can be mitigated by decomposition of the problem and parallel processing.

A limitation of our framework is also one that is shared by many other gene network inference procedures. Specifically that the simple network structure of a gene regulating another gene hides the true complexity of the transcriptional, translational and regulatory processes in the underlying biology, and it fails to provide mechanistic hypotheses for how a gene regulates other genes. Despite this limitation, this is a long-term goal that we and others in the field strive to attain by advancing these methods. Of particular note is that ODE methods have the inherent extensibility to incorporate more detailed and complex functional relationships that directly represent physical and causal mechanisms. The challenge is how to efficiently and correctly infer these functional relationships and associated parameters given the limited amount of biological data. We have hinted at one possibility for non-linear functions but considerable work still remains to improve the robustness and efficacy of these approaches.

While our framework presents a novel way to incorporate existing network information into the process of gene network inference, we believe it offers a more generic mechanism to incorporate other types of constraints. For example, research has indicated that different transcription factors have different occupancy levels in the promoter regions of the genes they regulate [20]. This might be modeled as constraints in our framework whereby transcription factors with low occupancy are given a high 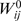 value while a high occupancy transcription factor is given a low 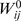 value. Of course this makes the assumption, possibly incorrectly, that the occupancy level has a proportional effect on transcription rate, but this may be an appropriate approximation for some systems. In the future, we look forward to investigating the many ways that we can incorporate the growing body of biological data into our optimization framework.

## Supporting Information

Appendix S1 Proofs of theorems
(0.20 MB DOC)

Text S1 Complete results for embryonic stem cell network
(0.08 MB DOC)
