# Supplementary material for: Incorporating Existing Network Information into Gene Network Inference
Source: PLoS One. 2009 Aug 27;4(8):e6799. doi: 10.1371/journal.pone.0006799 (PMC2729382; doi:10.1371/journal.pone.0006799)
Supplement: Appendix S1 — Proofs of theorems (0.20 MB DOC) [file pone.0006799.s001.doc]

# Appendix S1

## Proof of Theorems

Consider a matrix of non-negative entries and a parameter for L_1_-norm regularization:

(1.0)

This equation is equivalent to:

(1.1)

with matrices and defined to be:

(1.2)

Consider the following noise model:

(1.3)

where the noise term follows normal distribution with a fixed variance.

### Theorem 1

*If , and*

*(1.4)*

*is non-singular, then when ,*

*(1.5)*

*where*

*(1.6)*

*and is a random matrix with normal distribution of mean 0 and covariance for all i, j, k, l.*

*Proof.* Let . Then it follows from Eq. (1.1) that is the solution of

(1.7)

where

(1.8)

is a random matrix with mean 0 and covariance for all *a*, *b*, *c*, *d*. Here is the empirical covariance matrix of the input data. As . Consequently, we must have in Eq. (1.7) almost surely when *C* is non-singular. When , the last term in the RHS of Eq. (1.7) can be rewritten as

(1.9)

Now define . Then as minimizes

(1.10)

Given a matrix of non-negative entries and a single non-negative parameter , we can formulate the following minimization problem:

(1.11)

where is the component-wise product of the two matrices.

### Theorem 2

*If , and is non-singular, then*

*(1.12)*

*where*

*(1.13)*

*and Z is a random matrix with normal distribution of mean 0 and covariance for all i, j, k, l.*

*Proof.* Let . Then from Eq. (1.11) we have:

(1.14)

is a random variable. When , S follows normal distribution and converges in distribution to:

(1.15)

where Z follows normal distribution with mean 0 and covariance  for all . Hence, we have:

(1.16)

When almost surely. Thus, we can perform Taylor expansion on around which leads to:

(1.17)

When *Y* is small, the term can be rewritten as:

(1.18)

Substituting Eqs. (1.16), (1.17) and (1.18) into Eq. (1.14) and ignoring terms independent of *Y*, we have:

(1.19)

If we define , then we have .
