# Supplementary material for: Incorporating Existing Network Information into Gene Network Inference
Source: PLoS One. 2009 Aug 27;4(8):e6799. doi: 10.1371/journal.pone.0006799 (PMC2729382; doi:10.1371/journal.pone.0006799)
Supplement: Text S1 — Complete results for embryonic stem cell network (0.08 MB DOC) [file pone.0006799.s002.doc]

**Supplemental Text S1**

Scott Christley^1,2,3,4,§^, Qing Nie^1,3,4^ and Xiaohui Xie^2,3,4,5,§^

^1^Department of Mathematics, ^2^Department of Computer Science, ^3^Center for Mathematical and Computational Biology, ^4^Center for Complex Biological Systems, ^5^Institute for Genomics and Bioinformatics, University of California, Irvine, CA, USA

^§^Corresponding author

Email addresses:

SC: scott.christley@uci.edu

XX: xhx@uci.edu

QN: qnie@math.uci.edu

# The total list of 49 genes used for embryonic stem cell network

"71950:Nanog",

"18999:Pou5f1(Oct4)",

"20674:Sox2",

"16600:Klf4",

"58198:Sall1",

"99377:Sall4",

"51869:Rif1",

"75646:Rai14",

"11614:Nr0b1",

"19712:Rest",

"66830:Nacc1",

"19821:Rnf2",

"66932:Rexo1",

"67991:Nacc2",

"69890:Zfp219",

"56353:Rybp",

"13496:Arid3a",

"56380:Arid3b",

"20683:Sp1",

"14030:Ewsr1",

"226747:Ahctf1",

"105083:Pelo",

"226442:Zfp281",

"15182:Hdac2",

"21849:Trim28",

"15469:Prmt1",

"17869:Myc",

"22632:Yy1",

"218914:Wapal",

"216156:Wdr18",

"12534:Cdc2a",

"26380:Esrrb",

"21414:Tcf7",

"19664:Rbpj",

"104383:Rcor2",

"21420:Tcfap2c",

"20848:Stat3",

"21888:Tle4",

"18424:Otx2",

"13619:Phc1",

"26424:Nr5a2",

"104156:Etv5",

"13555:E2f1",

"22764:Zfx",

"81879:Tcfcp2l1",

"17125:Smad1",

"18109:Mycn",

"52615:Suz12",

"13018:Ctcf"

# Prior network from Zhou et al. [1]

26380:Esrrb 58198:Sall1 1

26380:Esrrb 13619:Phc1 1

26380:Esrrb 104156:Etv5 1

26380:Esrrb 56353:Rybp 1

26380:Esrrb 19664:Rbpj 1

26380:Esrrb 20674:Sox2 1

21414:Tcf7 58198:Sall1 1

21414:Tcf7 13619:Phc1 1

26424:Nr5a2 104156:Etv5 1

26424:Nr5a2 51869:Rif1 1

26424:Nr5a2 69890:Zfp219 1

26424:Nr5a2 56353:Rybp 1

26424:Nr5a2 21888:Tle4 1

26424:Nr5a2 18424:Otx2 1

26424:Nr5a2 13619:Phc1 1

26424:Nr5a2 58198:Sall1 1

26424:Nr5a2 19664:Rbpj 1

26424:Nr5a2 11614:Nr0b1 1

104156:Etv5 58198:Sall1 1

104156:Etv5 19664:Rbpj 1

104156:Etv5 11614:Nr0b1 1

104156:Etv5 56353:Rybp 1

99377:Sall4 58198:Sall1 1

20848:Stat3 20674:Sox2 1

20848:Stat3 13619:Phc1 1

20848:Stat3 21888:Tle4 1

21414:Tcf7 18424:Otx2 1

21414:Tcf7 18999:Pou5f1(Oct4) 1

71950:Nanog 26380:Esrrb 1

71950:Nanog 104156:Etv5 1

71950:Nanog 18999:Pou5f1(Oct4) 1

20674:Sox2 71950:Nanog 1

20674:Sox2 18999:Pou5f1(Oct4) 1

18999:Pou5f1(Oct4) 69890:Zfp219 1

18999:Pou5f1(Oct4) 71950:Nanog 1

20674:Sox2 18424:Otx2 1

18999:Pou5f1(Oct4) 18424:Otx2 1

20674:Sox2 51869:Rif1 1

18999:Pou5f1(Oct4) 51869:Rif1 1

20674:Sox2 19712:Rest 1

18999:Pou5f1(Oct4) 19712:Rest 1

20674:Sox2 104383:Rcor2 1

18999:Pou5f1(Oct4) 104383:Rcor2 1

20674:Sox2 21414:Tcf7 1

18999:Pou5f1(Oct4) 21414:Tcf7 1

71950:Nanog 19664:Rbpj 1

20674:Sox2 19664:Rbpj 1

18999:Pou5f1(Oct4) 19664:Rbpj 1

71950:Nanog 11614:Nr0b1 1

20674:Sox2 11614:Nr0b1 1

18999:Pou5f1(Oct4) 11614:Nr0b1 1

71950:Nanog 58198:Sall1 1

20674:Sox2 58198:Sall1 1

18999:Pou5f1(Oct4) 58198:Sall1 1

71950:Nanog 21420:Tcfap2c 1

20674:Sox2 21420:Tcfap2c 1

18999:Pou5f1(Oct4) 21420:Tcfap2c 1

71950:Nanog 21888:Tle4 1

20674:Sox2 21888:Tle4 1

18999:Pou5f1(Oct4) 21888:Tle4 1

71950:Nanog 13619:Phc1 1

20674:Sox2 13619:Phc1 1

18999:Pou5f1(Oct4) 13619:Phc1 1

71950:Nanog 56353:Rybp 1

20674:Sox2 56353:Rybp 1

18999:Pou5f1(Oct4) 56353:Rybp 1

# Prior network from Kim et al. [2]

71950:Nanog 71950:Nanog 1

20674:Sox2 71950:Nanog 1

11614:Nr0b1 71950:Nanog 1

66830:Nacc1 71950:Nanog 1

18999:Pou5f1(Oct4) 71950:Nanog 1

16600:Klf4 71950:Nanog 1

226442:Zfp281 71950:Nanog 1

17869:Myc 71950:Nanog 0

71950:Nanog 18999:Pou5f1(Oct4) 1

20674:Sox2 18999:Pou5f1(Oct4) 1

11614:Nr0b1 18999:Pou5f1(Oct4) 1

66830:Nacc1 18999:Pou5f1(Oct4) 1

18999:Pou5f1(Oct4) 18999:Pou5f1(Oct4) 1

16600:Klf4 18999:Pou5f1(Oct4) 1

226442:Zfp281 18999:Pou5f1(Oct4) 1

17869:Myc 18999:Pou5f1(Oct4) 0

71950:Nanog 20674:Sox2 1

20674:Sox2 20674:Sox2 1

11614:Nr0b1 20674:Sox2 1

66830:Nacc1 20674:Sox2 1

18999:Pou5f1(Oct4) 20674:Sox2 1

16600:Klf4 20674:Sox2 1

226442:Zfp281 20674:Sox2 1

17869:Myc 20674:Sox2 0

71950:Nanog 16600:Klf4 0

20674:Sox2 16600:Klf4 0

11614:Nr0b1 16600:Klf4 0

66830:Nacc1 16600:Klf4 0

18999:Pou5f1(Oct4) 16600:Klf4 0

16600:Klf4 16600:Klf4 1

226442:Zfp281 16600:Klf4 0

17869:Myc 16600:Klf4 0

71950:Nanog 99377:Sall4 1

20674:Sox2 99377:Sall4 1

11614:Nr0b1 99377:Sall4 1

66830:Nacc1 99377:Sall4 1

18999:Pou5f1(Oct4) 99377:Sall4 0

16600:Klf4 99377:Sall4 1

226442:Zfp281 99377:Sall4 0

17869:Myc 99377:Sall4 0

71950:Nanog 58198:Sall1 1

20674:Sox2 58198:Sall1 0

11614:Nr0b1 58198:Sall1 0

66830:Nacc1 58198:Sall1 1

18999:Pou5f1(Oct4) 58198:Sall1 1

16600:Klf4 58198:Sall1 1

226442:Zfp281 58198:Sall1 0

17869:Myc 58198:Sall1 0

71950:Nanog 51869:Rif1 1

20674:Sox2 51869:Rif1 1

11614:Nr0b1 51869:Rif1 1

66830:Nacc1 51869:Rif1 1

18999:Pou5f1(Oct4) 51869:Rif1 1

16600:Klf4 51869:Rif1 1

226442:Zfp281 51869:Rif1 0

17869:Myc 51869:Rif1 0

71950:Nanog 75646:Rai14 1

20674:Sox2 75646:Rai14 0

11614:Nr0b1 75646:Rai14 0

66830:Nacc1 75646:Rai14 1

18999:Pou5f1(Oct4) 75646:Rai14 1

16600:Klf4 75646:Rai14 0

226442:Zfp281 75646:Rai14 0

17869:Myc 75646:Rai14 0

71950:Nanog 11614:Nr0b1 1

20674:Sox2 11614:Nr0b1 1

11614:Nr0b1 11614:Nr0b1 1

66830:Nacc1 11614:Nr0b1 1

18999:Pou5f1(Oct4) 11614:Nr0b1 0

16600:Klf4 11614:Nr0b1 0

226442:Zfp281 11614:Nr0b1 0

17869:Myc 11614:Nr0b1 0

71950:Nanog 19712:Rest 1

20674:Sox2 19712:Rest 1

11614:Nr0b1 19712:Rest 1

66830:Nacc1 19712:Rest 1

18999:Pou5f1(Oct4) 19712:Rest 1

16600:Klf4 19712:Rest 1

226442:Zfp281 19712:Rest 0

17869:Myc 19712:Rest 0

71950:Nanog 19821:Rnf2 0

20674:Sox2 19821:Rnf2 0

11614:Nr0b1 19821:Rnf2 1

66830:Nacc1 19821:Rnf2 0

18999:Pou5f1(Oct4) 19821:Rnf2 0

16600:Klf4 19821:Rnf2 0

226442:Zfp281 19821:Rnf2 0

17869:Myc 19821:Rnf2 0

71950:Nanog 67991:Nacc2 0

20674:Sox2 67991:Nacc2 0

11614:Nr0b1 67991:Nacc2 1

66830:Nacc1 67991:Nacc2 0

18999:Pou5f1(Oct4) 67991:Nacc2 0

16600:Klf4 67991:Nacc2 0

226442:Zfp281 67991:Nacc2 0

17869:Myc 67991:Nacc2 0

71950:Nanog 69890:Zfp219 0

20674:Sox2 69890:Zfp219 0

11614:Nr0b1 69890:Zfp219 0

66830:Nacc1 69890:Zfp219 0

18999:Pou5f1(Oct4) 69890:Zfp219 1

16600:Klf4 69890:Zfp219 0

226442:Zfp281 69890:Zfp219 0

17869:Myc 69890:Zfp219 0

71950:Nanog 56353:Rybp 1

20674:Sox2 56353:Rybp 1

11614:Nr0b1 56353:Rybp 1

66830:Nacc1 56353:Rybp 0

18999:Pou5f1(Oct4) 56353:Rybp 0

16600:Klf4 56353:Rybp 1

226442:Zfp281 56353:Rybp 0

17869:Myc 56353:Rybp 1

71950:Nanog 13496:Arid3a 0

20674:Sox2 13496:Arid3a 0

11614:Nr0b1 13496:Arid3a 0

66830:Nacc1 13496:Arid3a 0

18999:Pou5f1(Oct4) 13496:Arid3a 0

16600:Klf4 13496:Arid3a 1

226442:Zfp281 13496:Arid3a 0

17869:Myc 13496:Arid3a 0

71950:Nanog 56380:Arid3b 1

20674:Sox2 56380:Arid3b 0

11614:Nr0b1 56380:Arid3b 0

66830:Nacc1 56380:Arid3b 0

18999:Pou5f1(Oct4) 56380:Arid3b 0

16600:Klf4 56380:Arid3b 0

226442:Zfp281 56380:Arid3b 0

17869:Myc 56380:Arid3b 0

71950:Nanog 20683:Sp1 0

20674:Sox2 20683:Sp1 0

11614:Nr0b1 20683:Sp1 0

66830:Nacc1 20683:Sp1 1

18999:Pou5f1(Oct4) 20683:Sp1 0

16600:Klf4 20683:Sp1 1

226442:Zfp281 20683:Sp1 0

17869:Myc 20683:Sp1 1

71950:Nanog 14030:Ewsr1 0

20674:Sox2 14030:Ewsr1 0

11614:Nr0b1 14030:Ewsr1 0

66830:Nacc1 14030:Ewsr1 0

18999:Pou5f1(Oct4) 14030:Ewsr1 0

16600:Klf4 14030:Ewsr1 1

226442:Zfp281 14030:Ewsr1 1

17869:Myc 14030:Ewsr1 1

17869:Myc 226747:Ahctf1 1

17869:Myc 226442:Zfp281 1

18999:Pou5f1(Oct4) 15182:Hdac2 1

226442:Zfp281 15182:Hdac2 1

17869:Myc 15182:Hdac2 1

226442:Zfp281 21849:Trim28 1

226442:Zfp281 15469:Prmt1 1

17869:Myc 15469:Prmt1 1

16600:Klf4 17869:Myc 1

17869:Myc 22632:Yy1 1

17869:Myc 218914:Wapal 1

17869:Myc 216156:Wdr18 1

17869:Myc 12534:Cdc2a 1

20674:Sox2 21414:Tcf7 1

18999:Pou5f1(Oct4) 21414:Tcf7 1

11614:Nr0b1 104383:Rcor2 1

66830:Nacc1 104383:Rcor2 1

71950:Nanog 21420:Tcfap2c 1

20674:Sox2 21420:Tcfap2c 1

11614:Nr0b1 21420:Tcfap2c 1

18999:Pou5f1(Oct4) 21420:Tcfap2c 1

16600:Klf4 21420:Tcfap2c 1

226442:Zfp281 21420:Tcfap2c 1

17869:Myc 21420:Tcfap2c 1

226442:Zfp281 20848:Stat3 1

17869:Myc 20848:Stat3 1

71950:Nanog 21888:Tle4 1

18999:Pou5f1(Oct4) 21888:Tle4 1

71950:Nanog 18424:Otx2 1

20674:Sox2 18424:Otx2 1

11614:Nr0b1 18424:Otx2 1

66830:Nacc1 18424:Otx2 1

16600:Klf4 18424:Otx2 1

226442:Zfp281 18424:Otx2 1

71950:Nanog 13619:Phc1 1

20674:Sox2 13619:Phc1 1

11614:Nr0b1 13619:Phc1 1

66830:Nacc1 13619:Phc1 1

18999:Pou5f1(Oct4) 13619:Phc1 1

16600:Klf4 13619:Phc1 1

71950:Nanog 104156:Etv5 1

20674:Sox2 104156:Etv5 1

11614:Nr0b1 104156:Etv5 1

18999:Pou5f1(Oct4) 104156:Etv5 1

71950:Nanog 81879:Tcfcp2l1 1

20674:Sox2 81879:Tcfcp2l1 1

11614:Nr0b1 81879:Tcfcp2l1 1

66830:Nacc1 81879:Tcfcp2l1 1

18999:Pou5f1(Oct4) 81879:Tcfcp2l1 1

16600:Klf4 81879:Tcfcp2l1 1

71950:Nanog 18109:Mycn 1

20674:Sox2 18109:Mycn 1

11614:Nr0b1 18109:Mycn 1

66830:Nacc1 18109:Mycn 1

226442:Zfp281 18109:Mycn 1

17869:Myc 52615:Suz12 1

18999:Pou5f1(Oct4) 13018:Ctcf 1

# Complete network with prior network information

71950:Nanog gr 18999:Pou5f1(Oct4)

71950:Nanog gr 20674:Sox2

71950:Nanog gr 16600:Klf4

71950:Nanog gr 58198:Sall1

71950:Nanog gr 11614:Nr0b1

71950:Nanog gr 19712:Rest

71950:Nanog gr 19821:Rnf2

71950:Nanog gr 67991:Nacc2

71950:Nanog gr 20683:Sp1

71950:Nanog gr 226747:Ahctf1

71950:Nanog gr 226442:Zfp281

71950:Nanog gr 21849:Trim28

71950:Nanog gr 17869:Myc

71950:Nanog gr 26380:Esrrb

71950:Nanog gr 19664:Rbpj

71950:Nanog gr 104383:Rcor2

71950:Nanog gr 21420:Tcfap2c

71950:Nanog gr 20848:Stat3

71950:Nanog gr 26424:Nr5a2

71950:Nanog gr 104156:Etv5

71950:Nanog gr 81879:Tcfcp2l1

71950:Nanog gr 18109:Mycn

71950:Nanog gr 52615:Suz12

18999:Pou5f1(Oct4) gr 71950:Nanog

18999:Pou5f1(Oct4) gr 20674:Sox2

18999:Pou5f1(Oct4) gr 16600:Klf4

18999:Pou5f1(Oct4) gr 58198:Sall1

18999:Pou5f1(Oct4) gr 99377:Sall4

18999:Pou5f1(Oct4) gr 11614:Nr0b1

18999:Pou5f1(Oct4) gr 19712:Rest

18999:Pou5f1(Oct4) gr 66830:Nacc1

18999:Pou5f1(Oct4) gr 19821:Rnf2

18999:Pou5f1(Oct4) gr 66932:Rexo1

18999:Pou5f1(Oct4) gr 67991:Nacc2

18999:Pou5f1(Oct4) gr 69890:Zfp219

18999:Pou5f1(Oct4) gr 56380:Arid3b

18999:Pou5f1(Oct4) gr 14030:Ewsr1

18999:Pou5f1(Oct4) gr 226747:Ahctf1

18999:Pou5f1(Oct4) gr 105083:Pelo

18999:Pou5f1(Oct4) gr 226442:Zfp281

18999:Pou5f1(Oct4) gr 15182:Hdac2

18999:Pou5f1(Oct4) gr 21849:Trim28

18999:Pou5f1(Oct4) gr 22632:Yy1

18999:Pou5f1(Oct4) gr 26380:Esrrb

18999:Pou5f1(Oct4) gr 19664:Rbpj

18999:Pou5f1(Oct4) gr 104383:Rcor2

18999:Pou5f1(Oct4) gr 21420:Tcfap2c

18999:Pou5f1(Oct4) gr 18424:Otx2

18999:Pou5f1(Oct4) gr 13619:Phc1

18999:Pou5f1(Oct4) gr 26424:Nr5a2

18999:Pou5f1(Oct4) gr 104156:Etv5

18999:Pou5f1(Oct4) gr 22764:Zfx

18999:Pou5f1(Oct4) gr 81879:Tcfcp2l1

18999:Pou5f1(Oct4) gr 17125:Smad1

18999:Pou5f1(Oct4) gr 52615:Suz12

18999:Pou5f1(Oct4) gr 13018:Ctcf

20674:Sox2 gr 71950:Nanog

20674:Sox2 gr 18999:Pou5f1(Oct4)

20674:Sox2 gr 99377:Sall4

20674:Sox2 gr 11614:Nr0b1

20674:Sox2 gr 67991:Nacc2

20674:Sox2 gr 226442:Zfp281

20674:Sox2 gr 19664:Rbpj

20674:Sox2 gr 21420:Tcfap2c

20674:Sox2 gr 18424:Otx2

20674:Sox2 gr 81879:Tcfcp2l1

20674:Sox2 gr 52615:Suz12

16600:Klf4 gr 71950:Nanog

16600:Klf4 gr 18999:Pou5f1(Oct4)

16600:Klf4 gr 11614:Nr0b1

16600:Klf4 gr 67991:Nacc2

16600:Klf4 gr 13496:Arid3a

16600:Klf4 gr 14030:Ewsr1

16600:Klf4 gr 226442:Zfp281

16600:Klf4 gr 17869:Myc

16600:Klf4 gr 21420:Tcfap2c

16600:Klf4 gr 81879:Tcfcp2l1

16600:Klf4 gr 52615:Suz12

58198:Sall1 gr 11614:Nr0b1

58198:Sall1 gr 67991:Nacc2

58198:Sall1 gr 226442:Zfp281

58198:Sall1 gr 52615:Suz12

99377:Sall4 gr 11614:Nr0b1

99377:Sall4 gr 19712:Rest

99377:Sall4 gr 67991:Nacc2

99377:Sall4 gr 13496:Arid3a

99377:Sall4 gr 56380:Arid3b

99377:Sall4 gr 226442:Zfp281

99377:Sall4 gr 104383:Rcor2

99377:Sall4 gr 52615:Suz12

51869:Rif1 gr 11614:Nr0b1

51869:Rif1 gr 67991:Nacc2

51869:Rif1 gr 226442:Zfp281

51869:Rif1 gr 52615:Suz12

75646:Rai14 gr 11614:Nr0b1

75646:Rai14 gr 19821:Rnf2

75646:Rai14 gr 67991:Nacc2

75646:Rai14 gr 226442:Zfp281

75646:Rai14 gr 19664:Rbpj

75646:Rai14 gr 22764:Zfx

75646:Rai14 gr 52615:Suz12

11614:Nr0b1 gr 71950:Nanog

11614:Nr0b1 gr 18999:Pou5f1(Oct4)

11614:Nr0b1 gr 67991:Nacc2

11614:Nr0b1 gr 226442:Zfp281

11614:Nr0b1 gr 21420:Tcfap2c

11614:Nr0b1 gr 81879:Tcfcp2l1

11614:Nr0b1 gr 52615:Suz12

19712:Rest gr 11614:Nr0b1

19712:Rest gr 19821:Rnf2

19712:Rest gr 67991:Nacc2

19712:Rest gr 20683:Sp1

19712:Rest gr 14030:Ewsr1

19712:Rest gr 226442:Zfp281

19712:Rest gr 22632:Yy1

19712:Rest gr 19664:Rbpj

19712:Rest gr 22764:Zfx

19712:Rest gr 52615:Suz12

66830:Nacc1 gr 18999:Pou5f1(Oct4)

66830:Nacc1 gr 11614:Nr0b1

66830:Nacc1 gr 67991:Nacc2

66830:Nacc1 gr 226442:Zfp281

66830:Nacc1 gr 81879:Tcfcp2l1

66830:Nacc1 gr 52615:Suz12

19821:Rnf2 gr 99377:Sall4

19821:Rnf2 gr 11614:Nr0b1

19821:Rnf2 gr 67991:Nacc2

19821:Rnf2 gr 226747:Ahctf1

19821:Rnf2 gr 226442:Zfp281

19821:Rnf2 gr 52615:Suz12

66932:Rexo1 gr 11614:Nr0b1

66932:Rexo1 gr 67991:Nacc2

66932:Rexo1 gr 226442:Zfp281

66932:Rexo1 gr 52615:Suz12

67991:Nacc2 gr 11614:Nr0b1

67991:Nacc2 gr 226442:Zfp281

67991:Nacc2 gr 52615:Suz12

69890:Zfp219 gr 11614:Nr0b1

69890:Zfp219 gr 67991:Nacc2

69890:Zfp219 gr 226442:Zfp281

69890:Zfp219 gr 52615:Suz12

56353:Rybp gr 11614:Nr0b1

56353:Rybp gr 67991:Nacc2

56353:Rybp gr 226442:Zfp281

56353:Rybp gr 52615:Suz12

13496:Arid3a gr 11614:Nr0b1

13496:Arid3a gr 67991:Nacc2

13496:Arid3a gr 226442:Zfp281

13496:Arid3a gr 52615:Suz12

56380:Arid3b gr 11614:Nr0b1

56380:Arid3b gr 67991:Nacc2

56380:Arid3b gr 226442:Zfp281

56380:Arid3b gr 52615:Suz12

20683:Sp1 gr 11614:Nr0b1

20683:Sp1 gr 67991:Nacc2

20683:Sp1 gr 226442:Zfp281

20683:Sp1 gr 22632:Yy1

20683:Sp1 gr 52615:Suz12

14030:Ewsr1 gr 11614:Nr0b1

14030:Ewsr1 gr 19712:Rest

14030:Ewsr1 gr 19821:Rnf2

14030:Ewsr1 gr 66932:Rexo1

14030:Ewsr1 gr 67991:Nacc2

14030:Ewsr1 gr 69890:Zfp219

14030:Ewsr1 gr 20683:Sp1

14030:Ewsr1 gr 226747:Ahctf1

14030:Ewsr1 gr 226442:Zfp281

14030:Ewsr1 gr 22632:Yy1

14030:Ewsr1 gr 21888:Tle4

14030:Ewsr1 gr 22764:Zfx

14030:Ewsr1 gr 52615:Suz12

14030:Ewsr1 gr 13018:Ctcf

226747:Ahctf1 gr 11614:Nr0b1

226747:Ahctf1 gr 67991:Nacc2

226747:Ahctf1 gr 226442:Zfp281

226747:Ahctf1 gr 52615:Suz12

105083:Pelo gr 11614:Nr0b1

105083:Pelo gr 67991:Nacc2

105083:Pelo gr 226442:Zfp281

105083:Pelo gr 52615:Suz12

226442:Zfp281 gr 18999:Pou5f1(Oct4)

226442:Zfp281 gr 11614:Nr0b1

226442:Zfp281 gr 67991:Nacc2

226442:Zfp281 gr 14030:Ewsr1

226442:Zfp281 gr 15182:Hdac2

226442:Zfp281 gr 21849:Trim28

226442:Zfp281 gr 21420:Tcfap2c

226442:Zfp281 gr 20848:Stat3

226442:Zfp281 gr 52615:Suz12

15182:Hdac2 gr 11614:Nr0b1

15182:Hdac2 gr 67991:Nacc2

15182:Hdac2 gr 226442:Zfp281

15182:Hdac2 gr 52615:Suz12

21849:Trim28 gr 16600:Klf4

21849:Trim28 gr 99377:Sall4

21849:Trim28 gr 11614:Nr0b1

21849:Trim28 gr 19712:Rest

21849:Trim28 gr 66830:Nacc1

21849:Trim28 gr 19821:Rnf2

21849:Trim28 gr 66932:Rexo1

21849:Trim28 gr 67991:Nacc2

21849:Trim28 gr 69890:Zfp219

21849:Trim28 gr 56353:Rybp

21849:Trim28 gr 13496:Arid3a

21849:Trim28 gr 56380:Arid3b

21849:Trim28 gr 20683:Sp1

21849:Trim28 gr 14030:Ewsr1

21849:Trim28 gr 105083:Pelo

21849:Trim28 gr 226442:Zfp281

21849:Trim28 gr 15469:Prmt1

21849:Trim28 gr 17869:Myc

21849:Trim28 gr 22632:Yy1

21849:Trim28 gr 216156:Wdr18

21849:Trim28 gr 12534:Cdc2a

21849:Trim28 gr 19664:Rbpj

21849:Trim28 gr 21420:Tcfap2c

21849:Trim28 gr 20848:Stat3

21849:Trim28 gr 21888:Tle4

21849:Trim28 gr 26424:Nr5a2

21849:Trim28 gr 13555:E2f1

21849:Trim28 gr 22764:Zfx

21849:Trim28 gr 18109:Mycn

21849:Trim28 gr 52615:Suz12

21849:Trim28 gr 13018:Ctcf

15469:Prmt1 gr 99377:Sall4

15469:Prmt1 gr 11614:Nr0b1

15469:Prmt1 gr 19712:Rest

15469:Prmt1 gr 66830:Nacc1

15469:Prmt1 gr 19821:Rnf2

15469:Prmt1 gr 67991:Nacc2

15469:Prmt1 gr 69890:Zfp219

15469:Prmt1 gr 13496:Arid3a

15469:Prmt1 gr 56380:Arid3b

15469:Prmt1 gr 20683:Sp1

15469:Prmt1 gr 14030:Ewsr1

15469:Prmt1 gr 226442:Zfp281

15469:Prmt1 gr 21849:Trim28

15469:Prmt1 gr 17869:Myc

15469:Prmt1 gr 22632:Yy1

15469:Prmt1 gr 20848:Stat3

15469:Prmt1 gr 21888:Tle4

15469:Prmt1 gr 52615:Suz12

17869:Myc gr 11614:Nr0b1

17869:Myc gr 67991:Nacc2

17869:Myc gr 14030:Ewsr1

17869:Myc gr 226442:Zfp281

17869:Myc gr 15182:Hdac2

17869:Myc gr 15469:Prmt1

17869:Myc gr 19664:Rbpj

17869:Myc gr 21420:Tcfap2c

17869:Myc gr 52615:Suz12

22632:Yy1 gr 11614:Nr0b1

22632:Yy1 gr 67991:Nacc2

22632:Yy1 gr 20683:Sp1

22632:Yy1 gr 226442:Zfp281

22632:Yy1 gr 19664:Rbpj

22632:Yy1 gr 52615:Suz12

218914:Wapal gr 11614:Nr0b1

218914:Wapal gr 67991:Nacc2

218914:Wapal gr 226442:Zfp281

218914:Wapal gr 52615:Suz12

216156:Wdr18 gr 11614:Nr0b1

216156:Wdr18 gr 67991:Nacc2

216156:Wdr18 gr 226442:Zfp281

216156:Wdr18 gr 52615:Suz12

12534:Cdc2a gr 11614:Nr0b1

12534:Cdc2a gr 67991:Nacc2

12534:Cdc2a gr 69890:Zfp219

12534:Cdc2a gr 13496:Arid3a

12534:Cdc2a gr 105083:Pelo

12534:Cdc2a gr 226442:Zfp281

12534:Cdc2a gr 22632:Yy1

12534:Cdc2a gr 19664:Rbpj

12534:Cdc2a gr 104383:Rcor2

12534:Cdc2a gr 52615:Suz12

26380:Esrrb gr 20674:Sox2

26380:Esrrb gr 16600:Klf4

26380:Esrrb gr 11614:Nr0b1

26380:Esrrb gr 67991:Nacc2

26380:Esrrb gr 13496:Arid3a

26380:Esrrb gr 20683:Sp1

26380:Esrrb gr 226442:Zfp281

26380:Esrrb gr 17869:Myc

26380:Esrrb gr 22632:Yy1

26380:Esrrb gr 20848:Stat3

26380:Esrrb gr 22764:Zfx

26380:Esrrb gr 52615:Suz12

21414:Tcf7 gr 18999:Pou5f1(Oct4)

21414:Tcf7 gr 11614:Nr0b1

21414:Tcf7 gr 67991:Nacc2

21414:Tcf7 gr 226442:Zfp281

21414:Tcf7 gr 52615:Suz12

19664:Rbpj gr 16600:Klf4

19664:Rbpj gr 58198:Sall1

19664:Rbpj gr 99377:Sall4

19664:Rbpj gr 11614:Nr0b1

19664:Rbpj gr 19712:Rest

19664:Rbpj gr 19821:Rnf2

19664:Rbpj gr 67991:Nacc2

19664:Rbpj gr 13496:Arid3a

19664:Rbpj gr 20683:Sp1

19664:Rbpj gr 226747:Ahctf1

19664:Rbpj gr 226442:Zfp281

19664:Rbpj gr 21849:Trim28

19664:Rbpj gr 17869:Myc

19664:Rbpj gr 22632:Yy1

19664:Rbpj gr 216156:Wdr18

19664:Rbpj gr 104383:Rcor2

19664:Rbpj gr 21420:Tcfap2c

19664:Rbpj gr 20848:Stat3

19664:Rbpj gr 13555:E2f1

19664:Rbpj gr 22764:Zfx

19664:Rbpj gr 52615:Suz12

19664:Rbpj gr 13018:Ctcf

104383:Rcor2 gr 11614:Nr0b1

104383:Rcor2 gr 67991:Nacc2

104383:Rcor2 gr 226442:Zfp281

104383:Rcor2 gr 52615:Suz12

21420:Tcfap2c gr 99377:Sall4

21420:Tcfap2c gr 11614:Nr0b1

21420:Tcfap2c gr 67991:Nacc2

21420:Tcfap2c gr 13496:Arid3a

21420:Tcfap2c gr 226442:Zfp281

21420:Tcfap2c gr 17869:Myc

21420:Tcfap2c gr 52615:Suz12

20848:Stat3 gr 18999:Pou5f1(Oct4)

20848:Stat3 gr 20674:Sox2

20848:Stat3 gr 99377:Sall4

20848:Stat3 gr 51869:Rif1

20848:Stat3 gr 75646:Rai14

20848:Stat3 gr 11614:Nr0b1

20848:Stat3 gr 19821:Rnf2

20848:Stat3 gr 67991:Nacc2

20848:Stat3 gr 69890:Zfp219

20848:Stat3 gr 56353:Rybp

20848:Stat3 gr 13496:Arid3a

20848:Stat3 gr 56380:Arid3b

20848:Stat3 gr 20683:Sp1

20848:Stat3 gr 14030:Ewsr1

20848:Stat3 gr 226747:Ahctf1

20848:Stat3 gr 105083:Pelo

20848:Stat3 gr 226442:Zfp281

20848:Stat3 gr 15182:Hdac2

20848:Stat3 gr 21849:Trim28

20848:Stat3 gr 15469:Prmt1

20848:Stat3 gr 22632:Yy1

20848:Stat3 gr 218914:Wapal

20848:Stat3 gr 216156:Wdr18

20848:Stat3 gr 12534:Cdc2a

20848:Stat3 gr 26380:Esrrb

20848:Stat3 gr 21414:Tcf7

20848:Stat3 gr 19664:Rbpj

20848:Stat3 gr 104383:Rcor2

20848:Stat3 gr 21888:Tle4

20848:Stat3 gr 13619:Phc1

20848:Stat3 gr 26424:Nr5a2

20848:Stat3 gr 22764:Zfx

20848:Stat3 gr 81879:Tcfcp2l1

20848:Stat3 gr 17125:Smad1

20848:Stat3 gr 18109:Mycn

20848:Stat3 gr 52615:Suz12

20848:Stat3 gr 13018:Ctcf

21888:Tle4 gr 11614:Nr0b1

21888:Tle4 gr 67991:Nacc2

21888:Tle4 gr 226442:Zfp281

21888:Tle4 gr 52615:Suz12

18424:Otx2 gr 11614:Nr0b1

18424:Otx2 gr 67991:Nacc2

18424:Otx2 gr 226442:Zfp281

18424:Otx2 gr 19664:Rbpj

18424:Otx2 gr 52615:Suz12

13619:Phc1 gr 16600:Klf4

13619:Phc1 gr 58198:Sall1

13619:Phc1 gr 11614:Nr0b1

13619:Phc1 gr 19712:Rest

13619:Phc1 gr 19821:Rnf2

13619:Phc1 gr 67991:Nacc2

13619:Phc1 gr 13496:Arid3a

13619:Phc1 gr 20683:Sp1

13619:Phc1 gr 14030:Ewsr1

13619:Phc1 gr 226442:Zfp281

13619:Phc1 gr 17869:Myc

13619:Phc1 gr 22632:Yy1

13619:Phc1 gr 12534:Cdc2a

13619:Phc1 gr 19664:Rbpj

13619:Phc1 gr 13555:E2f1

13619:Phc1 gr 22764:Zfx

13619:Phc1 gr 52615:Suz12

26424:Nr5a2 gr 11614:Nr0b1

26424:Nr5a2 gr 67991:Nacc2

26424:Nr5a2 gr 69890:Zfp219

26424:Nr5a2 gr 226442:Zfp281

26424:Nr5a2 gr 52615:Suz12

104156:Etv5 gr 11614:Nr0b1

104156:Etv5 gr 67991:Nacc2

104156:Etv5 gr 226442:Zfp281

104156:Etv5 gr 52615:Suz12

13555:E2f1 gr 11614:Nr0b1

13555:E2f1 gr 67991:Nacc2

13555:E2f1 gr 226442:Zfp281

13555:E2f1 gr 52615:Suz12

22764:Zfx gr 11614:Nr0b1

22764:Zfx gr 67991:Nacc2

22764:Zfx gr 226442:Zfp281

22764:Zfx gr 52615:Suz12

81879:Tcfcp2l1 gr 11614:Nr0b1

81879:Tcfcp2l1 gr 67991:Nacc2

81879:Tcfcp2l1 gr 226442:Zfp281

81879:Tcfcp2l1 gr 52615:Suz12

17125:Smad1 gr 11614:Nr0b1

17125:Smad1 gr 67991:Nacc2

17125:Smad1 gr 226442:Zfp281

17125:Smad1 gr 52615:Suz12

18109:Mycn gr 16600:Klf4

18109:Mycn gr 11614:Nr0b1

18109:Mycn gr 67991:Nacc2

18109:Mycn gr 226442:Zfp281

18109:Mycn gr 52615:Suz12

52615:Suz12 gr 16600:Klf4

52615:Suz12 gr 11614:Nr0b1

52615:Suz12 gr 19821:Rnf2

52615:Suz12 gr 67991:Nacc2

52615:Suz12 gr 20683:Sp1

52615:Suz12 gr 226747:Ahctf1

52615:Suz12 gr 226442:Zfp281

52615:Suz12 gr 22632:Yy1

52615:Suz12 gr 19664:Rbpj

13018:Ctcf gr 11614:Nr0b1

13018:Ctcf gr 67991:Nacc2

13018:Ctcf gr 226442:Zfp281

13018:Ctcf gr 52615:Suz12

# Complete network without prior network information

71950:Nanog gr 20674:Sox2

71950:Nanog gr 16600:Klf4

71950:Nanog gr 58198:Sall1

71950:Nanog gr 11614:Nr0b1

71950:Nanog gr 19712:Rest

71950:Nanog gr 19821:Rnf2

71950:Nanog gr 67991:Nacc2

71950:Nanog gr 13496:Arid3a

71950:Nanog gr 20683:Sp1

71950:Nanog gr 226747:Ahctf1

71950:Nanog gr 226442:Zfp281

71950:Nanog gr 21849:Trim28

71950:Nanog gr 26380:Esrrb

71950:Nanog gr 19664:Rbpj

71950:Nanog gr 104383:Rcor2

71950:Nanog gr 20848:Stat3

71950:Nanog gr 26424:Nr5a2

71950:Nanog gr 104156:Etv5

71950:Nanog gr 81879:Tcfcp2l1

71950:Nanog gr 52615:Suz12

18999:Pou5f1(Oct4) gr 71950:Nanog

18999:Pou5f1(Oct4) gr 20674:Sox2

18999:Pou5f1(Oct4) gr 16600:Klf4

18999:Pou5f1(Oct4) gr 58198:Sall1

18999:Pou5f1(Oct4) gr 99377:Sall4

18999:Pou5f1(Oct4) gr 11614:Nr0b1

18999:Pou5f1(Oct4) gr 19712:Rest

18999:Pou5f1(Oct4) gr 66830:Nacc1

18999:Pou5f1(Oct4) gr 19821:Rnf2

18999:Pou5f1(Oct4) gr 66932:Rexo1

18999:Pou5f1(Oct4) gr 67991:Nacc2

18999:Pou5f1(Oct4) gr 56380:Arid3b

18999:Pou5f1(Oct4) gr 226747:Ahctf1

18999:Pou5f1(Oct4) gr 105083:Pelo

18999:Pou5f1(Oct4) gr 226442:Zfp281

18999:Pou5f1(Oct4) gr 15182:Hdac2

18999:Pou5f1(Oct4) gr 21849:Trim28

18999:Pou5f1(Oct4) gr 22632:Yy1

18999:Pou5f1(Oct4) gr 26380:Esrrb

18999:Pou5f1(Oct4) gr 19664:Rbpj

18999:Pou5f1(Oct4) gr 104383:Rcor2

18999:Pou5f1(Oct4) gr 20848:Stat3

18999:Pou5f1(Oct4) gr 18424:Otx2

18999:Pou5f1(Oct4) gr 13619:Phc1

18999:Pou5f1(Oct4) gr 26424:Nr5a2

18999:Pou5f1(Oct4) gr 104156:Etv5

18999:Pou5f1(Oct4) gr 22764:Zfx

18999:Pou5f1(Oct4) gr 81879:Tcfcp2l1

18999:Pou5f1(Oct4) gr 52615:Suz12

18999:Pou5f1(Oct4) gr 13018:Ctcf

20674:Sox2 gr 99377:Sall4

20674:Sox2 gr 11614:Nr0b1

20674:Sox2 gr 67991:Nacc2

20674:Sox2 gr 226442:Zfp281

20674:Sox2 gr 19664:Rbpj

20674:Sox2 gr 52615:Suz12

16600:Klf4 gr 18999:Pou5f1(Oct4)

16600:Klf4 gr 11614:Nr0b1

16600:Klf4 gr 67991:Nacc2

16600:Klf4 gr 226442:Zfp281

16600:Klf4 gr 52615:Suz12

58198:Sall1 gr 11614:Nr0b1

58198:Sall1 gr 67991:Nacc2

58198:Sall1 gr 226442:Zfp281

58198:Sall1 gr 52615:Suz12

99377:Sall4 gr 18999:Pou5f1(Oct4)

99377:Sall4 gr 20674:Sox2

99377:Sall4 gr 11614:Nr0b1

99377:Sall4 gr 67991:Nacc2

99377:Sall4 gr 13496:Arid3a

99377:Sall4 gr 56380:Arid3b

99377:Sall4 gr 226442:Zfp281

99377:Sall4 gr 104383:Rcor2

99377:Sall4 gr 52615:Suz12

51869:Rif1 gr 11614:Nr0b1

51869:Rif1 gr 67991:Nacc2

51869:Rif1 gr 226442:Zfp281

51869:Rif1 gr 52615:Suz12

75646:Rai14 gr 11614:Nr0b1

75646:Rai14 gr 19821:Rnf2

75646:Rai14 gr 67991:Nacc2

75646:Rai14 gr 226442:Zfp281

75646:Rai14 gr 19664:Rbpj

75646:Rai14 gr 22764:Zfx

75646:Rai14 gr 52615:Suz12

11614:Nr0b1 gr 67991:Nacc2

11614:Nr0b1 gr 226442:Zfp281

11614:Nr0b1 gr 52615:Suz12

19712:Rest gr 11614:Nr0b1

19712:Rest gr 19821:Rnf2

19712:Rest gr 67991:Nacc2

19712:Rest gr 20683:Sp1

19712:Rest gr 226442:Zfp281

19712:Rest gr 22632:Yy1

19712:Rest gr 19664:Rbpj

19712:Rest gr 22764:Zfx

19712:Rest gr 52615:Suz12

66830:Nacc1 gr 11614:Nr0b1

66830:Nacc1 gr 67991:Nacc2

66830:Nacc1 gr 226442:Zfp281

66830:Nacc1 gr 52615:Suz12

19821:Rnf2 gr 99377:Sall4

19821:Rnf2 gr 11614:Nr0b1

19821:Rnf2 gr 67991:Nacc2

19821:Rnf2 gr 226747:Ahctf1

19821:Rnf2 gr 226442:Zfp281

19821:Rnf2 gr 22764:Zfx

19821:Rnf2 gr 52615:Suz12

19821:Rnf2 gr 13018:Ctcf

66932:Rexo1 gr 11614:Nr0b1

66932:Rexo1 gr 67991:Nacc2

66932:Rexo1 gr 226442:Zfp281

66932:Rexo1 gr 52615:Suz12

67991:Nacc2 gr 11614:Nr0b1

67991:Nacc2 gr 226442:Zfp281

67991:Nacc2 gr 52615:Suz12

69890:Zfp219 gr 11614:Nr0b1

69890:Zfp219 gr 67991:Nacc2

69890:Zfp219 gr 226442:Zfp281

69890:Zfp219 gr 52615:Suz12

56353:Rybp gr 11614:Nr0b1

56353:Rybp gr 67991:Nacc2

56353:Rybp gr 226442:Zfp281

56353:Rybp gr 52615:Suz12

13496:Arid3a gr 11614:Nr0b1

13496:Arid3a gr 67991:Nacc2

13496:Arid3a gr 226442:Zfp281

13496:Arid3a gr 52615:Suz12

56380:Arid3b gr 11614:Nr0b1

56380:Arid3b gr 67991:Nacc2

56380:Arid3b gr 226442:Zfp281

56380:Arid3b gr 52615:Suz12

20683:Sp1 gr 11614:Nr0b1

20683:Sp1 gr 67991:Nacc2

20683:Sp1 gr 226442:Zfp281

20683:Sp1 gr 22632:Yy1

20683:Sp1 gr 52615:Suz12

20683:Sp1 gr 13018:Ctcf

14030:Ewsr1 gr 20674:Sox2

14030:Ewsr1 gr 11614:Nr0b1

14030:Ewsr1 gr 19712:Rest

14030:Ewsr1 gr 19821:Rnf2

14030:Ewsr1 gr 66932:Rexo1

14030:Ewsr1 gr 67991:Nacc2

14030:Ewsr1 gr 20683:Sp1

14030:Ewsr1 gr 226747:Ahctf1

14030:Ewsr1 gr 226442:Zfp281

14030:Ewsr1 gr 22632:Yy1

14030:Ewsr1 gr 20848:Stat3

14030:Ewsr1 gr 21888:Tle4

14030:Ewsr1 gr 104156:Etv5

14030:Ewsr1 gr 22764:Zfx

14030:Ewsr1 gr 52615:Suz12

14030:Ewsr1 gr 13018:Ctcf

226747:Ahctf1 gr 11614:Nr0b1

226747:Ahctf1 gr 67991:Nacc2

226747:Ahctf1 gr 226442:Zfp281

226747:Ahctf1 gr 52615:Suz12

105083:Pelo gr 11614:Nr0b1

105083:Pelo gr 67991:Nacc2

105083:Pelo gr 226442:Zfp281

105083:Pelo gr 52615:Suz12

226442:Zfp281 gr 11614:Nr0b1

226442:Zfp281 gr 67991:Nacc2

226442:Zfp281 gr 52615:Suz12

15182:Hdac2 gr 11614:Nr0b1

15182:Hdac2 gr 67991:Nacc2

15182:Hdac2 gr 226442:Zfp281

15182:Hdac2 gr 52615:Suz12

21849:Trim28 gr 71950:Nanog

21849:Trim28 gr 18999:Pou5f1(Oct4)

21849:Trim28 gr 20674:Sox2

21849:Trim28 gr 16600:Klf4

21849:Trim28 gr 99377:Sall4

21849:Trim28 gr 11614:Nr0b1

21849:Trim28 gr 19712:Rest

21849:Trim28 gr 66830:Nacc1

21849:Trim28 gr 19821:Rnf2

21849:Trim28 gr 66932:Rexo1

21849:Trim28 gr 67991:Nacc2

21849:Trim28 gr 69890:Zfp219

21849:Trim28 gr 56353:Rybp

21849:Trim28 gr 56380:Arid3b

21849:Trim28 gr 20683:Sp1

21849:Trim28 gr 105083:Pelo

21849:Trim28 gr 226442:Zfp281

21849:Trim28 gr 15182:Hdac2

21849:Trim28 gr 15469:Prmt1

21849:Trim28 gr 17869:Myc

21849:Trim28 gr 22632:Yy1

21849:Trim28 gr 216156:Wdr18

21849:Trim28 gr 12534:Cdc2a

21849:Trim28 gr 19664:Rbpj

21849:Trim28 gr 20848:Stat3

21849:Trim28 gr 21888:Tle4

21849:Trim28 gr 18424:Otx2

21849:Trim28 gr 26424:Nr5a2

21849:Trim28 gr 104156:Etv5

21849:Trim28 gr 13555:E2f1

21849:Trim28 gr 22764:Zfx

21849:Trim28 gr 81879:Tcfcp2l1

21849:Trim28 gr 18109:Mycn

21849:Trim28 gr 52615:Suz12

21849:Trim28 gr 13018:Ctcf

15469:Prmt1 gr 20674:Sox2

15469:Prmt1 gr 99377:Sall4

15469:Prmt1 gr 11614:Nr0b1

15469:Prmt1 gr 19712:Rest

15469:Prmt1 gr 66830:Nacc1

15469:Prmt1 gr 19821:Rnf2

15469:Prmt1 gr 67991:Nacc2

15469:Prmt1 gr 13496:Arid3a

15469:Prmt1 gr 56380:Arid3b

15469:Prmt1 gr 20683:Sp1

15469:Prmt1 gr 226442:Zfp281

15469:Prmt1 gr 15182:Hdac2

15469:Prmt1 gr 21849:Trim28

15469:Prmt1 gr 17869:Myc

15469:Prmt1 gr 22632:Yy1

15469:Prmt1 gr 20848:Stat3

15469:Prmt1 gr 21888:Tle4

15469:Prmt1 gr 104156:Etv5

15469:Prmt1 gr 81879:Tcfcp2l1

15469:Prmt1 gr 52615:Suz12

15469:Prmt1 gr 13018:Ctcf

17869:Myc gr 11614:Nr0b1

17869:Myc gr 67991:Nacc2

17869:Myc gr 226442:Zfp281

17869:Myc gr 19664:Rbpj

17869:Myc gr 52615:Suz12

22632:Yy1 gr 11614:Nr0b1

22632:Yy1 gr 67991:Nacc2

22632:Yy1 gr 20683:Sp1

22632:Yy1 gr 226442:Zfp281

22632:Yy1 gr 19664:Rbpj

22632:Yy1 gr 52615:Suz12

218914:Wapal gr 11614:Nr0b1

218914:Wapal gr 67991:Nacc2

218914:Wapal gr 226442:Zfp281

218914:Wapal gr 52615:Suz12

216156:Wdr18 gr 11614:Nr0b1

216156:Wdr18 gr 67991:Nacc2

216156:Wdr18 gr 226442:Zfp281

216156:Wdr18 gr 52615:Suz12

12534:Cdc2a gr 20674:Sox2

12534:Cdc2a gr 11614:Nr0b1

12534:Cdc2a gr 67991:Nacc2

12534:Cdc2a gr 13496:Arid3a

12534:Cdc2a gr 105083:Pelo

12534:Cdc2a gr 226442:Zfp281

12534:Cdc2a gr 22632:Yy1

12534:Cdc2a gr 19664:Rbpj

12534:Cdc2a gr 104383:Rcor2

12534:Cdc2a gr 20848:Stat3

12534:Cdc2a gr 52615:Suz12

12534:Cdc2a gr 13018:Ctcf

26380:Esrrb gr 18999:Pou5f1(Oct4)

26380:Esrrb gr 20674:Sox2

26380:Esrrb gr 16600:Klf4

26380:Esrrb gr 11614:Nr0b1

26380:Esrrb gr 67991:Nacc2

26380:Esrrb gr 13496:Arid3a

26380:Esrrb gr 20683:Sp1

26380:Esrrb gr 226442:Zfp281

26380:Esrrb gr 21849:Trim28

26380:Esrrb gr 17869:Myc

26380:Esrrb gr 22632:Yy1

26380:Esrrb gr 20848:Stat3

26380:Esrrb gr 22764:Zfx

26380:Esrrb gr 52615:Suz12

21414:Tcf7 gr 11614:Nr0b1

21414:Tcf7 gr 67991:Nacc2

21414:Tcf7 gr 226442:Zfp281

21414:Tcf7 gr 52615:Suz12

19664:Rbpj gr 18999:Pou5f1(Oct4)

19664:Rbpj gr 20674:Sox2

19664:Rbpj gr 16600:Klf4

19664:Rbpj gr 58198:Sall1

19664:Rbpj gr 99377:Sall4

19664:Rbpj gr 11614:Nr0b1

19664:Rbpj gr 19712:Rest

19664:Rbpj gr 19821:Rnf2

19664:Rbpj gr 67991:Nacc2

19664:Rbpj gr 13496:Arid3a

19664:Rbpj gr 20683:Sp1

19664:Rbpj gr 226747:Ahctf1

19664:Rbpj gr 226442:Zfp281

19664:Rbpj gr 17869:Myc

19664:Rbpj gr 22632:Yy1

19664:Rbpj gr 216156:Wdr18

19664:Rbpj gr 104383:Rcor2

19664:Rbpj gr 21420:Tcfap2c

19664:Rbpj gr 20848:Stat3

19664:Rbpj gr 104156:Etv5

19664:Rbpj gr 13555:E2f1

19664:Rbpj gr 22764:Zfx

19664:Rbpj gr 81879:Tcfcp2l1

19664:Rbpj gr 52615:Suz12

19664:Rbpj gr 13018:Ctcf

104383:Rcor2 gr 11614:Nr0b1

104383:Rcor2 gr 67991:Nacc2

104383:Rcor2 gr 226442:Zfp281

104383:Rcor2 gr 52615:Suz12

21420:Tcfap2c gr 99377:Sall4

21420:Tcfap2c gr 11614:Nr0b1

21420:Tcfap2c gr 19712:Rest

21420:Tcfap2c gr 67991:Nacc2

21420:Tcfap2c gr 13496:Arid3a

21420:Tcfap2c gr 226442:Zfp281

21420:Tcfap2c gr 17869:Myc

21420:Tcfap2c gr 52615:Suz12

20848:Stat3 gr 18999:Pou5f1(Oct4)

20848:Stat3 gr 20674:Sox2

20848:Stat3 gr 99377:Sall4

20848:Stat3 gr 51869:Rif1

20848:Stat3 gr 75646:Rai14

20848:Stat3 gr 11614:Nr0b1

20848:Stat3 gr 19821:Rnf2

20848:Stat3 gr 67991:Nacc2

20848:Stat3 gr 69890:Zfp219

20848:Stat3 gr 56353:Rybp

20848:Stat3 gr 13496:Arid3a

20848:Stat3 gr 56380:Arid3b

20848:Stat3 gr 20683:Sp1

20848:Stat3 gr 14030:Ewsr1

20848:Stat3 gr 226747:Ahctf1

20848:Stat3 gr 105083:Pelo

20848:Stat3 gr 226442:Zfp281

20848:Stat3 gr 15182:Hdac2

20848:Stat3 gr 21849:Trim28

20848:Stat3 gr 15469:Prmt1

20848:Stat3 gr 22632:Yy1

20848:Stat3 gr 218914:Wapal

20848:Stat3 gr 216156:Wdr18

20848:Stat3 gr 12534:Cdc2a

20848:Stat3 gr 26380:Esrrb

20848:Stat3 gr 21414:Tcf7

20848:Stat3 gr 19664:Rbpj

20848:Stat3 gr 104383:Rcor2

20848:Stat3 gr 21420:Tcfap2c

20848:Stat3 gr 21888:Tle4

20848:Stat3 gr 18424:Otx2

20848:Stat3 gr 13619:Phc1

20848:Stat3 gr 26424:Nr5a2

20848:Stat3 gr 104156:Etv5

20848:Stat3 gr 22764:Zfx

20848:Stat3 gr 81879:Tcfcp2l1

20848:Stat3 gr 17125:Smad1

20848:Stat3 gr 52615:Suz12

20848:Stat3 gr 13018:Ctcf

21888:Tle4 gr 11614:Nr0b1

21888:Tle4 gr 67991:Nacc2

21888:Tle4 gr 226442:Zfp281

21888:Tle4 gr 52615:Suz12

18424:Otx2 gr 11614:Nr0b1

18424:Otx2 gr 67991:Nacc2

18424:Otx2 gr 226442:Zfp281

18424:Otx2 gr 19664:Rbpj

18424:Otx2 gr 52615:Suz12

13619:Phc1 gr 18999:Pou5f1(Oct4)

13619:Phc1 gr 16600:Klf4

13619:Phc1 gr 58198:Sall1

13619:Phc1 gr 11614:Nr0b1

13619:Phc1 gr 19712:Rest

13619:Phc1 gr 19821:Rnf2

13619:Phc1 gr 67991:Nacc2

13619:Phc1 gr 13496:Arid3a

13619:Phc1 gr 20683:Sp1

13619:Phc1 gr 226442:Zfp281

13619:Phc1 gr 22632:Yy1

13619:Phc1 gr 12534:Cdc2a

13619:Phc1 gr 19664:Rbpj

13619:Phc1 gr 104156:Etv5

13619:Phc1 gr 13555:E2f1

13619:Phc1 gr 22764:Zfx

13619:Phc1 gr 18109:Mycn

13619:Phc1 gr 52615:Suz12

26424:Nr5a2 gr 11614:Nr0b1

26424:Nr5a2 gr 67991:Nacc2

26424:Nr5a2 gr 226442:Zfp281

26424:Nr5a2 gr 52615:Suz12

104156:Etv5 gr 11614:Nr0b1

104156:Etv5 gr 67991:Nacc2

104156:Etv5 gr 226442:Zfp281

104156:Etv5 gr 52615:Suz12

13555:E2f1 gr 11614:Nr0b1

13555:E2f1 gr 67991:Nacc2

13555:E2f1 gr 226442:Zfp281

13555:E2f1 gr 52615:Suz12

22764:Zfx gr 11614:Nr0b1

22764:Zfx gr 67991:Nacc2

22764:Zfx gr 226442:Zfp281

22764:Zfx gr 52615:Suz12

81879:Tcfcp2l1 gr 11614:Nr0b1

81879:Tcfcp2l1 gr 67991:Nacc2

81879:Tcfcp2l1 gr 226442:Zfp281

81879:Tcfcp2l1 gr 52615:Suz12

17125:Smad1 gr 11614:Nr0b1

17125:Smad1 gr 67991:Nacc2

17125:Smad1 gr 226442:Zfp281

17125:Smad1 gr 52615:Suz12

18109:Mycn gr 16600:Klf4

18109:Mycn gr 11614:Nr0b1

18109:Mycn gr 67991:Nacc2

18109:Mycn gr 13496:Arid3a

18109:Mycn gr 226442:Zfp281

18109:Mycn gr 52615:Suz12

52615:Suz12 gr 20674:Sox2

52615:Suz12 gr 16600:Klf4

52615:Suz12 gr 11614:Nr0b1

52615:Suz12 gr 19821:Rnf2

52615:Suz12 gr 67991:Nacc2

52615:Suz12 gr 20683:Sp1

52615:Suz12 gr 226747:Ahctf1

52615:Suz12 gr 226442:Zfp281

52615:Suz12 gr 22632:Yy1

52615:Suz12 gr 19664:Rbpj

52615:Suz12 gr 81879:Tcfcp2l1

13018:Ctcf gr 18999:Pou5f1(Oct4)

13018:Ctcf gr 11614:Nr0b1

13018:Ctcf gr 67991:Nacc2

13018:Ctcf gr 226442:Zfp281

13018:Ctcf gr 52615:Suz12

# References

1. Zhou Q, Chipperfield H, Melton DA, Wong WH (2007) A gene regulatory network in mouse embryonic stem cells. Proc Natl Acad Sci USA. pp. 16438-16443.

2. Kim J, Chu J, Shen X, Wang J, Orkin SH (2008) An extended transcriptional network for pluripotency of embryonic stem cells. Cell. pp. 1049-1061.
